# Loss of FBXO9 Enhances Proteasome Activity and Promotes Aggressiveness in Acute Myeloid Leukemia

**DOI:** 10.3390/cancers11111717

**Published:** 2019-11-03

**Authors:** R. Willow Hynes-Smith, Samantha A. Swenson, Heather Vahle, Karli J. Wittorf, Mika Caplan, Catalina Amador, R. Katherine Hyde, Shannon M. Buckley

**Affiliations:** 1 Department of Genetics, Cell Biology, and Anatomy, University of Nebraska Medical Center, Omaha, NE 68198, USA; Rachel.hynes@unmc.edu (R.W.H.-S.); Samantha.swenson@unmc.edu (S.A.S.); heather.vahle@unmc.edu (H.V.); Karli.Wittorf@unmc.edu (K.J.W.); Mika.Calplan@huskers.unl.edu (M.C.); 2 Fred and Pamela Buffet Cancer Center, University of Nebraska Medical Center, Omaha, NE 68198, USA; catalina.amador@unmc.edu (C.A.); kate.hyde@unmc.edu (R.K.H.); 3 Department of Pathology and Microbiology, University of Nebraska Medical Center, Omaha, NE 68198, USA; 4 Department of Biochemistry and Molecular Biology, University of Nebraska Medical Center, Omaha, NE 68198, USA

**Keywords:** FBXO9, AML, bortezomib, proteasome, E3 ligase, F-Box

## Abstract

The hematopoietic system is maintained throughout life by stem cells that are capable of differentiating into all hematopoietic lineages. An intimate balance between self-renewal, differentiation, and quiescence is required to maintain hematopoiesis and disruption of this balance can result in malignant transformation. *FBXO9*, the substrate recognition component from the SCF E3 ubiquitin ligase family, is downregulated in patients with acute myeloid leukemia (AML) compared to healthy bone marrow, and this downregulation is particularly evident in patients with inv(16) AML. To study *FBXO9* in malignant hematopoiesis, we generated a conditional knockout mouse model using a novel CRISPR/Cas9 strategy. Deletion of *Fbxo9* in the murine hematopoietic system showed no adverse effects on stem and progenitor cell function but in AML lead to markedly accelerated and aggressive leukemia development in mice with inv(16). Not only did *Fbxo9* play a role in leukemia initiation but it also functioned to maintain AML activity and promote disease progression. Quantitative mass spectrometry from primary tumors reveals tumors lacking *Fbxo9* highly express proteins associated with metastasis and invasion as well as components of the ubiquitin proteasome system. We confirmed that the loss of *FBXO9* leads to increased proteasome activity and tumors cells were more sensitive to in vitro proteasome inhibition with bortezomib, suggesting that *FBXO9* expression may predict patients’ response to bortezomib.

## 1. Introduction

Acute myeloid leukemia (AML) is a hematologic malignancy resulting in an accumulation of immature myeloid blasts that impair normal hematopoiesis [1]. This disease accounts for 35% of new leukemia diagnoses and 48% of leukemia-related deaths [2]. In 2019, AML was estimated to be the most frequently diagnosed leukemia and the only common form with a higher mortality rate than incidence. Although new therapies have recently been approved, they are limited to specific subtypes of AML and only increase the treatment options for limited subsets of patients [3,4,5,6]. To develop more effective and less toxic therapies for use across multiple subtypes, we must better understand the mechanisms underlying the development and progression of AML.

One system that has not been extensively studied in the context of AML is the ubiquitin proteasome system (UPS). The UPS coordinates the degradation of proteins globally and compartmentally within a cell and is a key regulatory mechanism for many cellular processes, including cell cycle, transcription, and proliferation [7]. Two main components of this system are the E3 ubiquitin ligases that determine substrate ubiquitin modification and the 26S proteasome responsible for protein degradation. This system has proven to be a viable target in hematologic malignances, either through targeting the 26S proteasome with drugs such as bortezomib [8] or by altering substrate recognition of E3 ligases, as is done with thalidomide [9]. The successful utilization of these drugs in other hematologic malignancies suggests that targeting the UPS in AML could prove effective in treating the disease.

Ubiquitin E3 ligases can be classified as RING-finger, HECT-domain, or RBR, based on their domains and mode of transferring ubiquitin to the substrate [10]. The largest family of E3 ligases, the Skp1-Cul1-F-box (SCF) family, is composed of a core complex, including S-phase kinase-associated protein 1 (Skp1) and Cullin 1 (Cul1), which are scaffolding proteins that bring the ubiquitin binding RING-finger protein Rbx in proximity with the substrate recognition F-box protein component [11]. There are 69 F-box proteins that interact with Skp1 via the F-box domain, and with substrate proteins through a variety of substrate-recognition domains [12,13]. Dysregulation of E3 ligases, including many F-box proteins, has been correlated with aberrant hematopoiesis and malignant transformation [14,15]. A number of proteins from the F-box family have been classified as tumor suppressors, others as oncogenes, and a third set with context-specific roles [16]. FBXW7, for example, plays an integral role in hematopoietic stem and progenitor cell (HSPC) self-renewal, and its loss has been linked to drug-resistant T-cell acute lymphoblastic leukemia, whereas loss of chronic myeloid leukemia (CML) inhibits the initiation and progression of disease [17,18,19]. Loss of FBXO4 increases extramedullary myeloid hematopoiesis and is highly expressed in various lymphomas [20]. Other family members have been linked to leukemia cell proliferation, including FBXL2, FBXL10, FBXW11, and SKP2 [21,22,23,24]. Furthermore, overexpressed *FBXO9* in multiple myeloma (MM) degraded TEL2 and TTI1, shifting signaling from mTORC1 to mTORC2, thus causing increased proliferation and survival [25].

In this study, we identified *FBXO9* as an important regulator of AML and found it has low expression in patients across all AML subtypes. To study the role of *Fbxo9* in AML, we developed a conditional knockout (cKO) mouse model and monitored the leukemia response in vivo. We utilized a mass spectrometry (MS)-based approach, to identify proteins upregulated when *Fbxo9* expression is lost in tumors and identified various proteins previously shown to participate in cancer-related mechanisms, such as metastasis, proliferation, invasion, and metabolism. Notably, we found that many upregulated proteins participate in proteasome-regulated pathways. Furthermore, our in vitro analysis found that cKO tumors had increased proteasome activity and responded better to bortezomib treatment. Our studies provide insight into the role of the UPS in AML and present evidence that selecting patients according to *FBXO9* expression could be used as a method of identifying tumors to treat with proteasome inhibitors.

## 2. Results

### 2.1. FBXO9 Has Low Expression in AML and Expression Correlates to Poor Survival

**Figure 1:**
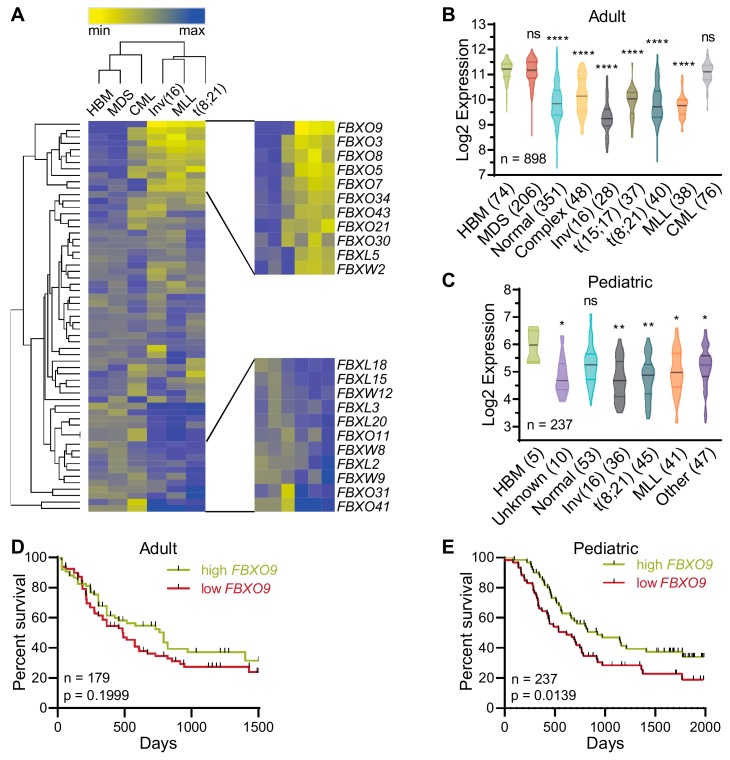
*FBXO9* expression is reduced in acute myeloid leukemia (AML) patients and correlates with poor survival. (**A**) Of all F-box family proteins, *FBXO9* has the lowest expression in select AML subtypes. (**B**,**C**) Analysis of patient samples from the (**B**) MILE and (**C**) pediatric TARGET studies reveals that AML patients have low *FBXO9* expression across a variety of subtypes when compared to healthy bone marrow (BM)—number of patients per subtype in parentheses. (**D**,**E**) Poor survival correlates with low *FBXO9* expression in both (**D**) adults and (**E**) children (ns = statistically non-significant, * *p* < 0.05, ** *p* < 0.01, **** *p* < 0.0001).

To identify F-box proteins involved in the initiation and/or progression of AML, we analyzed patient data from the Microarray Innovations in LEukemia (MILE) study for expression of 61 F-box proteins (GSE13159) [26]. The MILE study included microarray expression analyses from 898 patients, including 28 inv(16), 38 MLL-rearranged (MLL), 40 t(8;21), 37 t(15;17), 48 complex karyotype, and 351 normal karyotype AML patients. Analysis revealed that *FBXO9* has the lowest expression in inv(16), MLL-rearranged (MLL), and t(8;21) AMLs among the F-box proteins. Additionally, when compared to healthy bone marrow (HBM), CML, and myelodysplastic syndrome (MDS), *FBXO9* showed decreased expression (Figure 1A and Figure S1A). Further analysis across a wider variety of AML subtypes, including normal and complex karyotype and t(15;17), revealed that *FBXO9* is consistently downregulated across all subtypes (Figure 1B). In particular, patients with inv(16) AML had significantly reduced expression when compared to the other subtypes (normal, *p* = 0.0002; complex, *p* < 0.0001; t(15;17), *p* = 0.0004; t(8;21), *p* = 0.0086; MLL, *p* = 0.0036). As AML is the second most common childhood leukemia, we utilized the TARGET pediatric study of 237 pediatric AML patients to analyze *FBXO9* expression and again found downregulation of *FBXO9* in all AML subtypes, except patients with normal karyotype (Figure 1C) [27]. *FBXO9* expression in inv(16) AML within the pediatric patients is not significantly different from the other subtypes, with the exception of those with normal karyotype (*p* = 0.0006) and unknown (*p* = 0.0118).

Correlation of patient expression versus survival revealed that adult patients with FBXO9 expression below the median (lower 50% of patients) tend to have a worse prognosis and shorter time of survival compared to patients with expression above the median (top 50% of patients), particularly within the first 1500 days post-diagnosis (Figure 1D and Figure S1B) [28]. While the survival difference in the smaller cohort of adult patients only approaches significance, the larger cohort of pediatric patients demonstrates that within the first 2000 days post-diagnosis, low *FBXO9* expression correlated with poor survival (Figure 1E). Patients who went into remission and survived over 1500 (adult) or 2000 (pediatric) days post initial diagnosis had no significant difference in survival nor was the difference significant in any one subtype, though they trended toward poor prognosis with low *FBXO9* (Figure S1C–H) [28]. Taken together, these findings suggest that *FBXO9* expression is decreased in AML cells, and that low expression correlates with poor survival at early time-points from initial diagnosis.

### 2.2. Generation of Conditional Knockout of Fbxo9

**Figure 2:**
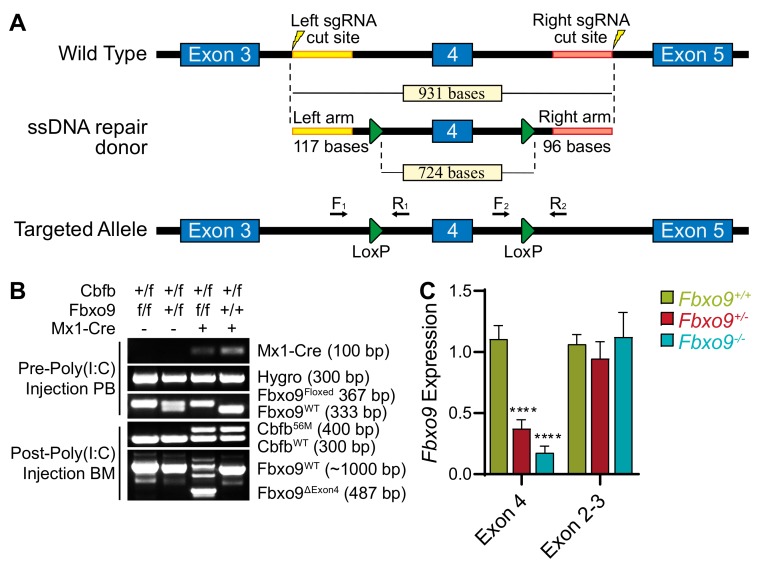
Generation of conditional *Fbxo9* knockout mouse model. (**A**) Using the Easi-CRISPR method, LoxP sites were introduced into the introns flanking *Fbxo9* exon 4. (**B**) Genotyping PCR demonstrates the introduction of LoxP sites, the presence of the *Cbfb^+/56M^* transgenic allele, and shows the loss of exon 4 and expression of the *Cbfb-MYH11* fusion gene, following injections with Poly(I:C). (**C**) *Fbxo9* exon 4 cKO is confirmed by qRT-PCR analysis, while exons 2–3 remain undisturbed (**** *p* < 0.0001).

Considering that *FBXO9* expression is reduced in AML patients and that this reduction correlates with a negative prognosis, we generated a cKO mouse to provide a model for studying the role of *FBXO9* in the tumor-initiating population, and in the development and progression of AML. *FBXO9* has two known domains: the F-box domain for binding the SCF complex, and the protein-binding TPR domain [11]. Using the Easi-CRISPR method, we introduced LoxP sites flanking *Fbxo9* exon 4, which contains the majority of the TPR domain (Figure 2A) [29]. The targeted mouse was bred with an Mx1-cre mouse. In mice expressing the floxed *Fbxo9* and Mx1-cre alleles, the cre recombinase is activated in the hematopoietic system through administration of Polyinosinic:Polycytidilic acid (Poly(I:C)), and deletes *Fbxo9* exon 4 [30].

We confirmed the genotypes by PCR and analyzed *Fbxo9* expression in replicate *Fbxo9^+/−^* and *Fbxo9^−/−^* mice, compared to *Fbxo9^+/+^*. The expression within the BM of our cKO groups was reduced ~55% in *Fbxo9^+/−^* and ~80% in *Fbxo9^−/−^* (Figure 2B,C). Loss of exon 4 results in ablation of TPR binding and initiates a frame–shift mutation, leading to the additional loss of the F-box domain and a premature stop that was confirmed by PCR and sequencing in *Fbxo9^−/−^* BM (Figure S2A). Due to lack of a commercially available antibody recognizing mouse *FBXO9*, we transfected 293T cells with tagged *Fbxo9* mutant plasmids, including a mutant lacking exon 4 to mimic the mutation that arises in the mouse model following Poly(I:C) injection. Mutants lacking only the F-box or the TPR domain showed a slight decrease in molecular weight, whereas the mutant lacking bases corresponding to exon 4 showed a much greater decrease in molecular weight (Figure S2B). The band size correlated with the estimated molecular weight of a protein arising from cKO cells predicted to have a frameshift mutation leading to a premature stop and loss of F-box domain. The evidence indicates that deletion of exon 4 in our mouse model results in a truncated protein lacking the TPR and F-box domains.

### 2.3. Fbxo9 Deletion Leads to Alterations in HSPC Populations

**Figure 3:**
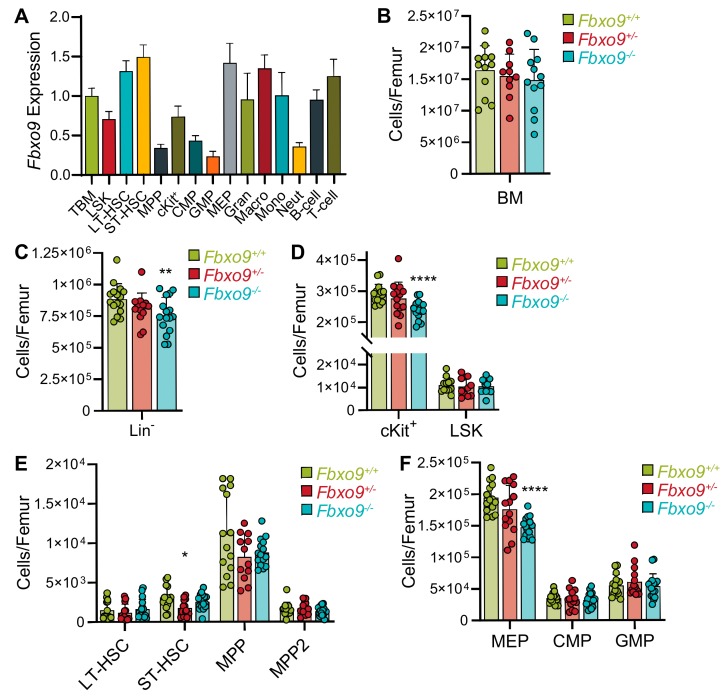

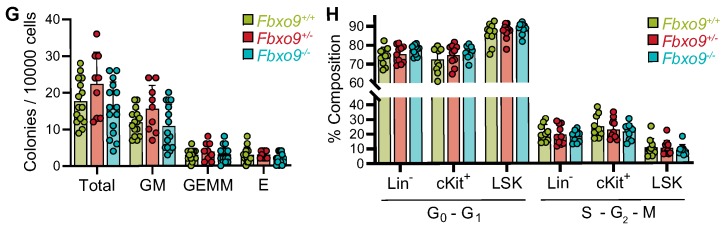
Loss of *Fbxo9* alters murine HSPCs. (**A**) Analysis of *Fbxo9* expression in WT murine hematopoietic lineages shows expression differences in a variety of hematopoietic lineages (TBM, total bone marrow; LSK, Lin^−^cKit^+^Sca-1^+;^ LT-HSC, Lin^−^cKit^+^Sca-1^+^CD150^+^CD48^−^; ST-HSC, Lin^−^cKit^+^Sca-1^+^CD150^−^CD48^−^; MPP, multipotent progenitors, Lin^−^cKit^+^Sca-1^+^CD48^+^; cKit^+^, Lin^−^cKit^+^Sca-1^-^; CMP, common myeloid progenitor, Lin^−^cKit^+^Sca-1^-^CD34^+^CD16/32^lo^; GMP, granulocyte-macrophage progenitor, Lin^−^cKit^+^Sca-1^-^CD34^+^CD16/32^hi^; MEP, Lin^−^cKit^+^Sca-1^−^CD34^−^CD16/32^lo^); gran, granulocyte, CD11b^-^Gr1^+^; macro, macrophages, CD11b^+^Gr1^−^; mono, monocyte, CD11b^+^Gr1^lo^; neut, neutrophil, CD11b^+^Gr1^hi^). (**B**) Bar graph of cell counts from BM extracted from the right femur of sacrificed mice. (**C**–**F**) Bar graphs showing the cell count of Lin^−^, cKit^+^, LSKs, LT-HSC, ST-HSC, two MPP (MPP, Lin^−^cKit^+^Sca-1^+^CD150^−^CD48^+^ and MPP2, Lin^−^cKit^+^Sca-1^+^CD150^+^CD48^+^), GMP, CMP, and MEP compartments in the BM of mice of the indicated genotypes. (**G**) Bar graph of colonies per 10000 cells plated in methyl cellulose (GM, granulocyte-macrophage; GEMM, granulocyte-erythrocyte-monocyte-megakaryocyte; E, erythroid). (**H**) Bar graph showing the percentages of cells in G_0_-G_1_ and S-G_2_-M (for all data shown, bar graphs are mean ± standard deviation, *n* = 3, * *p* < 0.05, ** *p* < 0.01, **** *p* < 0.0001).

By analyzing different hematopoietic populations isolated from BM of a healthy WT mouse, we found that *Fbxo9* is most highly expressed in short- and long-term hematopoietic stem cells (ST- and LT-HSCs), as well as some of the myeloid lineages (Figure 3A). To study the role of *Fbxo9* in normal hematopoiesis, we deleted *Fbxo9* from cKO mice by three sequential injections with Poly(I:C). Flow cytometry analysis of the BM four weeks post-Poly(I:C) revealed a slight, though not significant, decrease in total cell number upon knockout of *Fbxo9* (Figure 3B). Though we observed no difference in the distribution of mature hematopoietic populations (myeloid, T lymphoid, or B lymphoid), the lineage negative (Lin^−^) cells, which include HPSCs, showed a moderate decrease in total number upon loss of *Fbxo9,* corresponding to the marginal decrease in total cell number (Figure 3C and Figure S3A). Further analysis of the HSPCs revealed that *Fbxo9* cKO did not affect the stem and early progenitor cells (LSK) but caused a decrease in the cKit^+^ progenitor population (cKit^+^), due to a loss of megakaryocyte-erythroid progenitors (MEP) (Figure 3D–F). To determine whether loss of *Fbxo9* alters differentiation of HSPCs, we carried out a colony-forming cell assay and found that the proliferative capacity of LSKs derived from all three genotypes remained unaltered in response to cytokine stimulation (Figure 3G). We further analyzed these populations for apoptosis and cell cycle alterations and found that *Fbxo9* cKO alters neither the survival nor cell cycle progression of Lin^−^, cKit^+^, and LSK cells (Figure 3H and Figure S3B,C). To analyze the effect loss of *Fbxo9* has on the ability of HSCs to engraft and proliferate, we conducted a competitive repopulation assay and found that loss of *Fbxo9* neither impairs nor enhances their proliferative capacity and ability to engraft (Figure S3D). Together, these results indicate that, though highly expressed in HSC populations, loss of *Fbxo9* does not adversely affect HSPC maintenance and differentiation.

### 2.4. Deletion of Fbxo9 Leads to an Aggressive and Immature AML Phenotype

The lowest expression of *FBXO9* was found in the inv(16) subtype of AML. Inv(16)/t(16;16) arises from an inversion or translocation within chromosome 16 that fuses the genes for core binding factor beta (*Cbfb*) and smooth muscle myosin heavy chain (*MYH11*) [31]. The resulting CBFβ-SMMHC fusion protein alters Runt-related protein 1 (RUNX1, formerly AML1) activity, a transcription factor important in hematopoietic regulation, which leads to a block in the differentiation of myeloid progenitor cells [32,33]. To study the role of *Fbxo9* in inv(16) AML, we crossed our *Mx^Cre^Fbxo9^f/f^* mouse with the floxed allele of *Cbfb-MYH11* (*Cbfb^+/56M^*), which allows for the inducible expression of CBFβ-SMMHC following administration of Poly(I:C) [32]. In mice, expression of the *Cbfb^+/56M^* allele is sufficient to initiate AML development with a median survival of approximately 20 weeks [32]. We confirmed the presence of *Cbfb^56M^* within the offspring prior to Poly(I:C) treatment and confirmed deletion of exon 4 and expression of *Cbfb-MYH11* following Poly(I:C) by PCR (Figure 2B).

**Figure 4:**
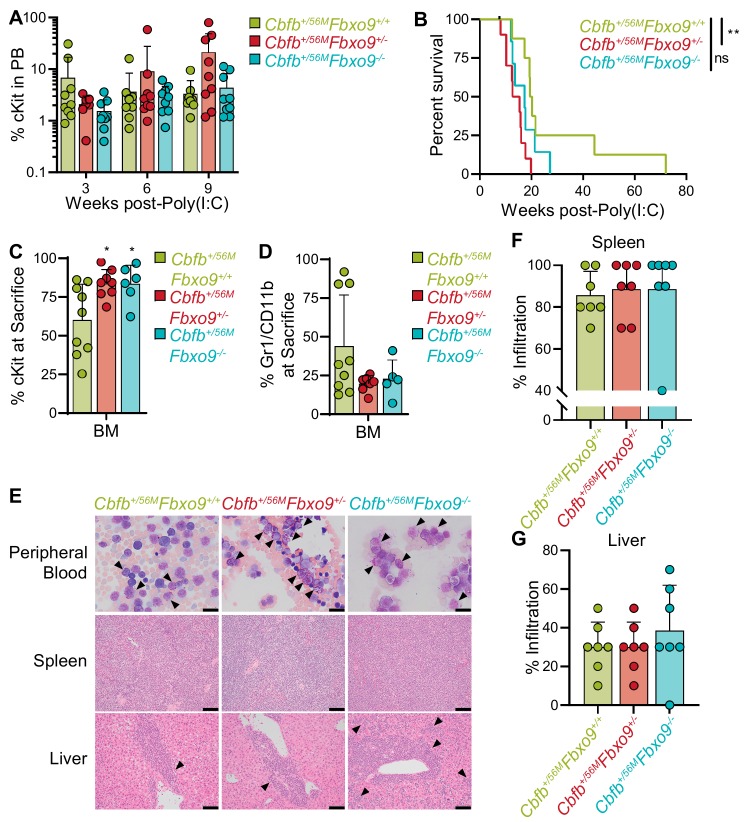
Loss of *Fbxo9* accelerates inv(16) AML and causes a more aggressive disease phenotype. (**A**) Analysis of the PB of mice following initiation of inv(16) AML (mean ± standard deviation). (**B**) Kaplan–Meier survival curves of Poly(I:C) treated mice (*n* = 8). (**C**,**D**) Bar graphs (mean ± standard deviation) representing the percentage of (**C**) cKit^+^ tumor cells or (**D**) Gr1^+^/CD11b^+^ cells in the BM of mice at time of sacrifice. (**E**) Representative images of Wright–Giemsa-stained peripheral blood and hematoxylin-eosin (H&E)-stained spleen and liver sections of mice with the indicated genotypes at time of sacrifice. Bars, 20 µm. (**F**,**G**) Quantification of the infiltration in the (**F**) spleen (*Cbfb^+/56M^Fbxo9^+/−^*, *p* = 0.6751 and *Cbfb^+/56M^Fbxo9^−/−^*, *p* = 0.7647) and (**G**) liver (*Cbfb^+/56M^Fbxo9^+/−^*, *p* > 0.9999 and *Cbfb^+/56M^Fbxo9^−/−^*, *p* = 0.4128), where 100% represents infiltration and complete effacement while 0% represents no infiltration with normal tissue architecture (*n* = 7) (ns = statistically non-significant, * *p* < 0.05, ** *p* < 0.01).

To monitor disease onset, we performed flow cytometry on peripheral blood (PB), beginning three weeks post-Poly(I:C) injection. Cell surface markers for mature myeloid cells, Gr1 and CD11b, were analyzed along with cKit to identify immature AML blasts. Analysis revealed that *Cbfb^+/56M^Fbxo9^+/+^* mice tend to have an early (two to three weeks) expression of cKit^+^ cells, that diminishes at weeks six and nine. Although not significantly different, the kinetics of the cKit^+^ tumor population following *Fbxo9* deletion differ, and this population expands at weeks six and nine (Figure 4A). Upon signs of terminal illness including white toes, hunched posture, sluggish movement, and palpable spleen, mice were sacrificed and analyzed to examine disease state. We found that the mice developed AML within the expected latency period, though the *Cbfb^+/56M^Fbxo9^+/−^* group had a significantly shorter time of survival with a median survival of 14 weeks (*p* = 0.0037) compared to 17 weeks (*p* = 0.0731) in *Cbfb^+/56M^Fbxo9^−/−^,* and 20 weeks in *Cbfb^+/56M^Fbxo9^+/+^* cohorts (Figure 4B). Although the *Cbfb^+/56M^Fbxo9^−/−^* cohort did not show significant change in survival a larger cohort may reveal an acceleration of disease initiation.

Consistent with previously reported data, ~80% of mice in the *Cbfb^+/56M^Fbxo9^+/+^* control group had tumors with a predominantly blast-like population, expressing the cell surface marker cKit, and ~20% expressed the more mature cell surface markers Gr1 and CD11b [32]. Upon deletion of *Fbxo9,* all of the mice from both *Cbfb^+/56M^Fbxo9^−/−^* and *Cbfb^+/56M^Fbxo9^+/−^* cohorts developed blast-like tumors, expressing cKit on the cell surface and lacking Gr1/CD11b expression, suggesting a more immature phenotype (Figure 4C,D and Figure S4A). Consequently, cKO mice had a greater expression of cKit in BM upon sacrifice, indicating a greater tumor burden in the BM upon the loss of one or both *Fbxo9* alleles (Figure 4C). These data showed that *Cbfb^+/56M^Fbxo9^+/−^* results in a shorter time of survival, and both *Cbfb^+/56M^Fbxo9^+/−^* and *Cbfb^+/56M^Fbxo9^−/−^* give rise to tumors with a more immature phenotype.

In addition, we analyzed secondary organs for signs of infiltration. The spleens from all groups had splenomegaly (Figure S4B). H&E staining of spleen infiltration demonstrated that ~60% of *Cbfb^+/56M^Fbxo9^−/−^* mice had complete effacement of the spleen architecture compared to only 30% of *Cbfb^+/56M^Fbxo9^+/+^* mice (Figure 4E,F). Likewise, in the liver, *Cbfb^+/56M^Fbxo9^−/−^* mice had ~50% infiltration in half the cohort, indicating a more aggressive disease, even though they had a similar time of survival to *Cbfb^+/56M^Fbxo9^+/+^* mice (Figure 4G). These findings suggest that loss of *Fbxo9* leads to increased infiltration of spleen and liver.

### 2.5. Transplanted Tumors with Reduced Fbxo9 Lead to Rapid Onset of Disease

**Figure 5:**
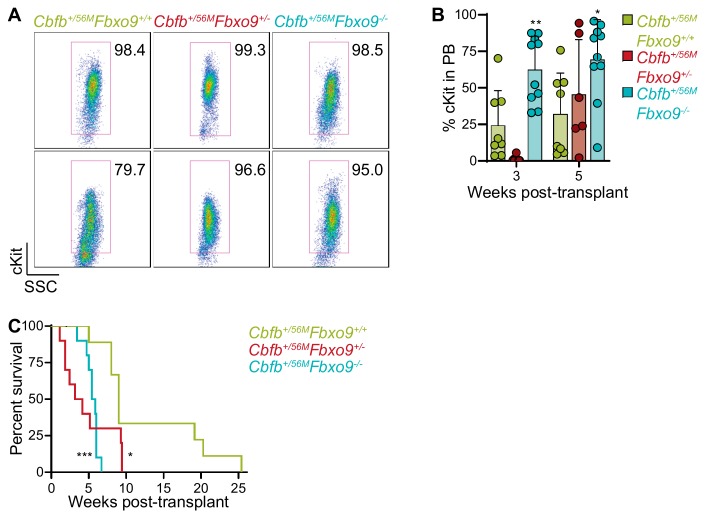
Transplant of inv(16) spleen tumor cells with lower expression of *Fbxo9* develop more aggressively. (**A**) FACS plots of spleen tumor cells transplanted into sub-lethally irradiated host mice. (**B**) Bar graph (mean ± standard deviation) representing the percentage of cKit^+^ cells in the peripheral blood (PB) of transplant mice. (**C**) Overall survival of mice transplanted with tumors of the indicated genotypes (*n* = 10, * *p* < 0.05, ** *p* < 0.01, *** *p* < 0.001).

Development of primary AML tumors demonstrated that loss of *Fbxo9* alters the initiation and phenotype of inv(16) AML. To determine whether *Fbxo9* is acting only in a tumor-initiating fashion or if it also has a role in tumor maintenance, we carried out a secondary transplantation. Tumor cells from spleens were injected into sub-lethally irradiated recipient mice. To eliminate bias from tumor phenotypes, we selected primary splenic tumor cells with a cKit^+^ phenotype (Figure 5A). All tumors selected contained > 95% tumor burden with the exception of one *Cbfb^+/56M^Fbxo9^+/+^* tumor, with only 80% cKit^+^ spleen cells. This tumor was selected due to its aggressive nature that resulted in the shortest survival from the *Cbfb^+/56M^Fbxo9^+/+^* cohort.

We followed the development of AML and by five weeks post-transplant, *Cbfb^+/56M^Fbxo9^−/−^* mice had on average 69.6% tumor PB compared to only 25% in the *Cbfb^+/56M^Fbxo9^+/+^,* indicating that tumors with decreased *Fbxo9* develop more rapidly (Figure 5B.) We also observed an earlier effect on normal hematopoiesis, as seen in the significant decrease in B220^+^ cells in the PB of the *Cbfb^+/56M^Fbxo9^+/−^* and *Cbfb^+/56M^Fbxo9^−/−^* mice at only three weeks post-transplant (Figure S5A). The swift expansion of the tumor population and rapid impairment of normal hematopoiesis led to a decreased time of survival in *Cbfb^+/56M^Fbxo9^+/−^* and *Cbfb^+/56M^Fbxo9^−/−^* transplant cohorts, compared to *Cbfb^+/56M^Fbxo9^+/+^* (Figure 5C). Once again, mice were sacrificed upon signs of terminal illness in accordance with IACUC standards. Upon sacrifice, analysis of the BM, spleen, and PB by flow cytometry showed no difference in tumor burden between the three cohorts (Figure S5B). Though no difference was observed in spleen infiltration, the infiltration in the livers of the transplant mice was higher in the *Cbfb^+/56M^Fbxo9^+/+^* cohort than in either of the cKO cohorts (Figure S5C,D). The variation in infiltration likely stems from the length of survival time. The more aggressive cKO tumors caused such a rapid progression of the disease in the BM and spleen that they didn’t have sufficient time to infiltrate the liver to the degree of the *Cbfb^+/56M^Fbxo9^+/^,^+^* tumors, which developed more slowly. Secondary transplantation confirms that tumors lacking *Fbxo9* are aggressive, and that this protein is involved in the maintenance and progression of inv(16) AML.

### 2.6. Loss of Fbxo9 Leads to Upregulation of Proteins Associated with Tumorigenicity

**Figure 6:**
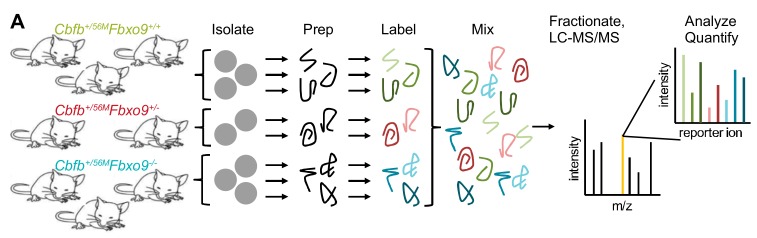

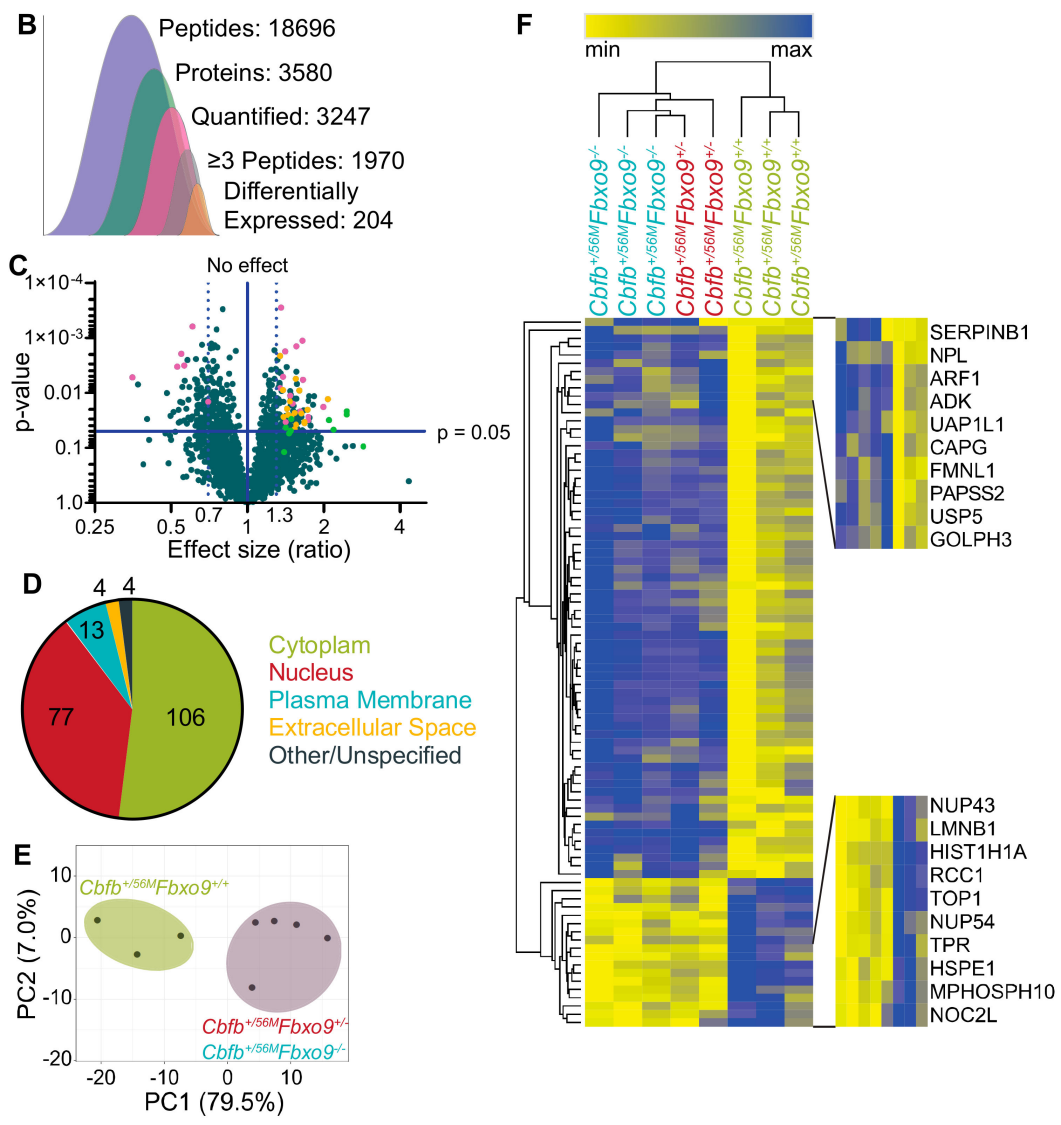
Tumors lacking *Fbxo9* are more similar than tumors with WT expression. (**A**) Schematic of preparation for tandem mass tag (TMT) MS using tumors isolated from *Cbfb^+/56M^Fbxo9^+/+^*, *Cbfb^+/56M^Fbxo9^+/−^*, and *Cbfb^+/56M^Fbxo9^−/−^* mice (*n* = 3). (**B**) Quantification of the identified peptides and proteins. (**C**) Volcano plot of the fold change *Cbfb^+/56M^Fbxo9^−/−^/Cbfb^+/56M^Fbxo9^+/+^* samples for significantly upregulated and significantly downregulated proteins (pink, significantly upregulated in *Cbfb^+/56M^Fbxo9^+/−^/Cbfb^+/56M^Fbxo9^+/+^*; yellow, proteins participating in proteasome-dependent pathways; green, top upregulated proteins associated with tumorigenicity). (**D**) Pie chart of localizations for the differentially expressed proteins identified in either the *Cbfb^+/56M^Fbxo9^+/−^*, *Cbfb^+/56M^Fbxo9^−/−^*, or both cohorts. (**E**) PCA plot using components 1 and 2, showing clustering of *Cbfb^+/56M^Fbxo9^+/+^* tumors compared to *Cbfb^+/56M^Fbxo9^+/−^* and *Cbfb^+/56M^Fbxo9^−/−^* tumors. (**F**) Heatmap with hierarchical clustering of the significantly upregulated (*p* < 0.05, ≥1.3 fold increase over WT) and downregulated (p < 0.05, ≤0.7 fold decrease from WT) proteins.

To identify potential *Fbxo9* substrates and protein alterations in AML, we performed quantitative MS on splenic tumor cells. We labeled isolated proteins with tandem mass tags, combined equal concentrations from each sample, and analyzed by MS (LC-MS/MS) (Figure 6A). Mass spectrometry identified 18,696 unique peptides representing 3580 proteins, 3247 of which were quantifiable (Figure 6B). To quantify protein changes, we compared only proteins with at least three unique peptides. Between *Cbfb^+/56M^Fbxo9^+/−^* and *Cbfb^+/56M^Fbxo9^−/−^* cohorts, 118 proteins were significantly upregulated (*p* < 0.05, fold change ≥ 1.3) and 86 proteins were significantly downregulated (*p* < 0.05, fold change ≤ 0.7) in one or both cohorts (Figure 6C, Table S1).

The majority of these differentially expressed proteins were localized in the cytoplasm where *FBXO9* is expressed (Figure 6D). To determine whether tumors with different genotypes show distinct patterns of expression, we carried out a principle component analysis (PCA) and hierarchical clustering and found that three independent tumors with WT expression of *Fbxo9* clustered together while heterozygous and homozygous cKO tumors clustered together, indicating they are more similar to each other than to *Cbfb^+/56M^Fbxo9^+/+^* tumors (Figure 6E,F). Interestingly, a number of the top upregulated proteins identified (SERPINB1, ADK, ARF1, UAP1L1, CAPG, FMNL1, PAPSS2, USP5, and GOLPH3) have been shown to participate in cancer by increasing cell growth and metastasis, or are biomarkers for poor outcomes (Figure 6F) [34,35,36,37,38,39,40,41,42].

### 2.7. Loss of Fbxo9 Correlates with Increased Proteasome Activity

**Figure 7:**
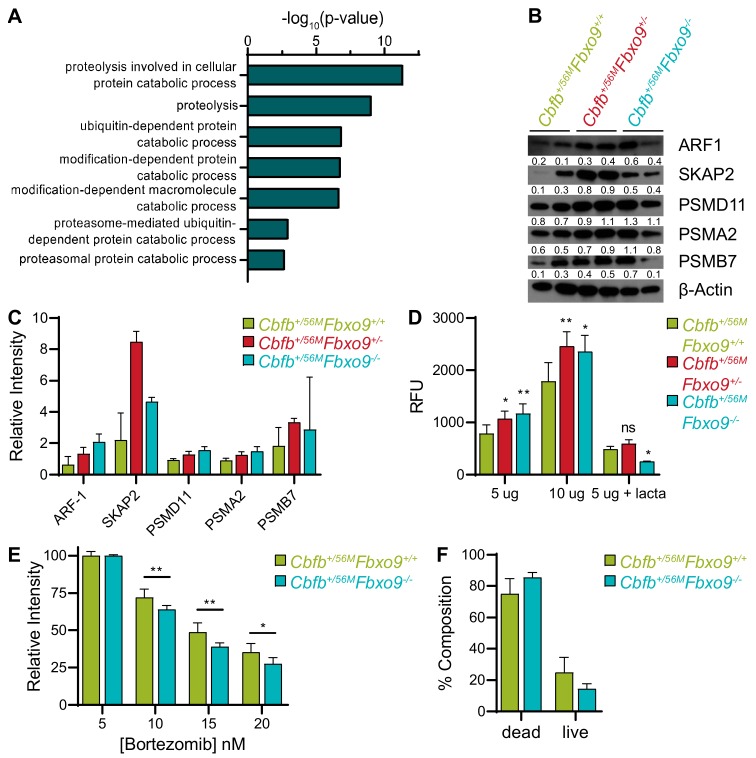
Knockout of *Fbxo9* results in an increase in proteasome activity and differing response to proteasome inhibition in vitro. (**A**) Gene ontology analysis showing pathways known to be associated with proteins significantly upregulated (*p* < 0.05, ≥1.3 fold change), using DAVID 6.8 software. For upregulated proteins, pathways included have ≥10 associated proteins and *p* < 0.01. (**B**) Western blot analysis of identified upregulated proteins and proteasome components for expression in murine inv(16) AML tumors with the indicated genotypes. (**C**) Bar graph quantifying relative expression of the proteins compared to β-actin expression. (**D**) Bar graph of proteasome activity in cultured tumor cells using the indicated amounts of protein, proteasome activity confirmed by treatment with the proteasome inhibitor lactacystin. (**E**) Bar graph showing survival of *Cbfb^+/56M^Fbxo9^+/+^* and *Cbfb^+/56M^Fbxo9^−/−^* tumor cells after 24 h culture with increasing doses of bortezomib. (**F**) Bar graph quantifying cell death of tumors cultured 16 h with 20 nM bortezomib, cell death analyzed by flow cytometry using Annexin-V and 7AAD; for live/dead, *p* = 0.1502 (for all data shown *n* = 3, all bar graphs are mean ± standard deviation, * *p* < 0.05, ** *p* < 0.01).

The proteins overexpressed upon loss of *Fbxo9* were also enriched for proteins associated with proteasome-mediated pathways, such as proteolysis and ubiquitin- or proteasome-dependent catabolism (Figure 7A). Increased proteasome activity has previously been implicated in cancer aggression and, accordingly, proteasome inhibitors have been approved as a treatment [43]. Considering this strong correlation between cancer and proteasome activity, we confirmed proteasome component overexpression by western blot analysis. We observed an increase in differentially expressed proteasome components in tumors lacking *Fbxo9*, though some variation was seen between the different tumor samples (Figure 7B,C and Figure S6). Patient RNA expression data revealed no correlation of *FBXO9* with *ARF1, PSMA2*, *PSMB7*, or *PSMD11,* and only a marginal positive correlation with *SKAP2* (Figure S7A–E). Furthermore, when broken down into AML subtypes, we observed no significant increase of RNA, as would be expected if regulation of these proteins is occurring during transcription (Figure S7F–J). These findings suggest that RNA expression of *FBXO9* does not correlate with RNA expression of *ARF1, PSMA2*, *PSMB7*, and *PSMD11,* and these genes are not differentially expressed at the RNA level in AML. However, these findings do not account for protein expression in AML patients, and high protein expression in tumors lacking *Fbxo9* suggests that elevated protein expression leads to aggressive AML phenotypes in mice.

To validate that proteasome component expression correlated with increased activity *in vitro*, we performed a proteasome activity assay comparing our tumor groups. This confirmed that not only did loss of *Fbxo9* result in increased proteasome component expression, but that this expression correlated with increased proteasome activity in the *Cbfb^+/56M^Fbxo9^+/−^* and *Cbfb^+/56M^Fbxo9^−/−^* tumors (Figure 7D). To further elucidate the effect of loss of *Fbxo9* in AML, we treated cultured tumor cells with varying concentrations of the proteasome inhibitor bortezomib (Figure 7E). Cells treated for 24 h with bortezomib confirmed that *Cbfb^+/56M^Fbxo9^−/−^* tumor cells were more sensitive to proteasome inhibition than *Cbfb^+/56M^Fbxo9^+/+^* tumor cells, with IC_50_ calculations of 10.03 nM and 11.76 nM, respectively. Analysis of cell death by flow cytometry after 16 h bortezomib treatment demonstrated that *Cbfb^+/56M^Fbxo9^−/−^* tumor cells trended toward increased sensitivity compared to *Cbfb^+/56M^Fbxo9^+/+^* tumor cells, though a longer treatment time is required to achieve statistical significance (Figure 7F). These studies provide evidence that the loss of *Fbxo9* leads to increased proteasome activity and sensitivity to proteasome inhibitors like bortezomib.

## 3. Discussion

The molecular pathogenesis of AML has yet to be fully defined, though many acquired molecular and cytogenetic abnormalities have been identified that lead to leukemogenesis [44]. The majority of these alterations are thought to occur in the HSPCs and disease progression is often thought to progress through the dysregulation of normal cellular mechanisms [45]. As *FBXO9* is an E3 ligase highly expressed in HSCs (the tumor-initiating population), we first explored the possibility that loss of its expression would result in changes to HSPC function that might lead to development of AML. We report that loss of *Fbxo9* correlates with a marginal decrease in the Lin^−^ cells of the BM, that primarily comes from a decrease in the cKit^+^ progenitor cells within that compartment. Further analyses determined that the loss of *Fbxo9* expression within the hematopoietic system has little to no effect on HSPC maintenance and differentiation. We saw no alterations in cell cycle, survival, or in the distribution of mature hematopoietic cells in the BM, despite observing a decrease in cKit^+^ cells. This observed change could be due to altered quiescence or rate of differentiation and further investigation into the mechanistic changes that occur in HSPCs with diminished *Fbxo9* expression could reveal an important role in hematopoiesis. To understand the mechanistic changes associated with disease initiation and progression, however, it is essential to determine the role FBXO9 plays in the development and progression of AML.

Aberrant *FBXO9* expression has previously been linked to disease progression in MM, by tagging mTORC1 components for degradation [25]. In this context, overexpression was shown to promote disease progression and, thus, *FBXO9* has previously been classified as an oncogene. Contrary to its role in MM, our studies demonstrate that *FBXO9* expression is consistently decreased across AML subtypes and that reduction of its E3 ligase activity promotes the progression of AML and tends to decrease survival time, both in mouse studies and patient cohorts. Additionally, MS experiments did not show accumulation of the known substrates TEL2 and TTI1, identified in MM, indicating that it has an alternative mechanism of action in leukemia. This finding demonstrates that *FBXO9* plays a context-specific role in cancer, acting as an oncogene in MM and a tumor suppressor in AML. While many other F-box proteins have been shown to have solely oncogenic or tumor suppressor roles, *FBXO9* is among a few select members of the family that can function in both capacities [16]. Furthermore, we found that loss of a single copy of *Fbxo9* was sufficient to cause increased aggressiveness in AML tumor cells. Considering that *FBXO9* is an E3 ligase, we must elucidate the alterations that the loss of this protein causes in the proteomic landscape of AML cells.

Through interrogation of the proteomic changes occurring in murine AML tumor cells upon loss of *Fbxo9*, we identified various proteasome components and proteasome-related proteins that were upregulated. Increased proteasome activity has been associated with aggressiveness in many cancer types, through in vitro and in vivo studies [46,47,48,49]. The work of Ma et al. reported that AML patients had higher levels of 20S proteasome components compared to healthy controls but this increase did not lead to increased chymotrypsin-like activity due to a reduction in the expression of the 19S regulatory component [50]. Similarly, our data showed increased proteasome component expression of the 20S catalytic subunit components (PSMA and PSMB) in *Cbfb^+/56M^Fbxo9^−/−^,* compared to *Cbfb^+/56M^Fbxo9^+/+^*. Unlike the results produced by Ma et al., we saw increased expression in 19S (PSMD) components, indicating that loss of *Fbxo9* could correspond with altered proteasome activity. Further analysis revealed that increased component expression did, indeed, lead to increased proteasome activity, suggesting a direct association between the two.

Proteasome inhibition with drugs such as bortezomib has become the standard of care for patients with MM and has been effectively utilized as a second-line therapy in treating mantle cell lymphoma and follicular lymphoma [51,52,53]. Clinical trials using bortezomib to treat AML, either alone or as a combination drug, have reported varying complete remission rates ranging from 0% to 80% [54,55,56,57,58,59,60]. To date, no specific AML mutation or classification has been correlated with patient response to proteasome inhibition. Indeed, responses seem to be independent of AML subtype. Furthermore, one of the main impediments to achieving complete remission in AML stems from the inability of current therapies to target the leukemic stem cells. Bortezomib has the additional benefit of being more effective in targeting leukemic stem cells, cells that rely heavily upon proteasome activity for survival [61,62].

Proteasome inhibitors have previously been effective in targeting the tumor-initiating population and mature tumor cells and the findings presented herein could lead to more efficacious clinical use of agents like bortezomib. In general, bortezomib is well-tolerated and has minimal side effects in adults [63], with the exception of patients hypersensitive to bortezomib, boron, or mannitol. The Children’s Oncology Group conducted a randomized trial in 1097 pediatric patients with AML, who they treated with standard chemotherapy with or without the addition of bortezomib [64]. They found that bortezomib did not improve overall event-free survival in children but had increased peripheral neuropathy, contraindicating the addition of bortezomib to the treatment regimen of pediatric patients with de novo AML [64]. Therapeutic use of bortezomib to treat AML may not be suitable for all adult patients but in those for whom first-line therapies prove ineffective, bortezomib could provide the treatment needed to improve patient outcome, particularly with improved methods of predicting patient response. Overall, we have identified *Fbxo9* as a tumor suppressor of AML and shown that loss of its expression leads to increased proteasome activity and sensitivity to proteasome inhibition, thus implying that *FBXO9* expression could be used as a biomarker to identify patients who would respond well to proteasome inhibition.

## 4. Materials and Methods

### 4.1. Patient Datasets

*FBXO9* expression and survival analyses in adult and pediatric patients are derived from the Microarray Innovations in Leukemia (MILE) study [26], the Therapeutically Applicable Research to Generate Effective Treatments (TARGET) [27], and the Genomic and Epigenomic Landscapes of Adult De Novo Acute Myeloid Leukemia [28]. Patient statistics can be found in Supplemental Materials and methods. Heatmap generation was carried out by calculating the Z-score for each individual F-box, in order to make comparisons between subtypes and among the different F-boxes. Hierarchal clustering was conducted using Euclidean distance. Violin plots were generated by plotting the log2 RNA expression for each patient within the various subtypes.

### 4.2. Transgenic Mouse Models

*Fbxo9* cKO mice were developed using Easi-CRISPR, as previously published [29], and bred with *Cbfb^+/56M^* [29,32] or Mx-cre mice, purchased from Jackson Laboratories (#003556, Bar Harbor, ME, USA). PCR confirmed the genotype and the expression of these genes (primers listed in Supplemental Materials and Methods). To induce cKO of *Fbxo9* and expression of *Cbfb-MYH11*, 6–8 week-old floxed mice and littermate controls received three intraperitoneal Poly(I:C) injections every other day, at a dose of 10 µg per gram body weight (Invivogen, San Diego, CA, USA). Procedures performed were approved by the Institutional Animal Care and Use Committee of the University of Nebraska Medical Center in accordance with NIH guidelines (protocol number: 15-099-11-FC).

### 4.3. Flow Cytometry Analysis

For flow cytometry analyses, PB was extracted from the tail vein and RBCs were lysed with 500 µL ACK lysing buffer. Upon sacrifice, spleen and BM cells were strained through 0.45 µm strainer and treated with ACK lysing buffer. Spleen, BM, and PB cells were stained for 1 h on ice in the dark, in 3% FBS in PBS (antibodies listed in Supplemental Materials and methods). For cell cycle analysis, cells were fixed and permeabilized following Biolegend intracellular staining protocol, and stained with Ki67 and DAPI, using 10 µL DAPI per sample.

### 4.4. Colony Forming Cell (CFC) Assay

Fresh or culture progeny from TBM were counted and 5000 cells/well of a 24-well plate were resuspended in Methocult (M3434, Stem Cell Technologies, Vancouver, BC, Canada). Colonies were counted at day 10, resuspended, and replated as before.

### 4.5. Histological Staining

Mouse organs were fixed in 10% (vol/vol) buffered formalin phosphate for 24 h and stored in 70% ethanol. Sections were stained with H&E using standard protocols. The slides for each treatment group were evaluated and graded by a pathologist for leukemia infiltration.

### 4.6. Western Blot Analysis

For Western blot analysis, samples were lysed in an IP lysis buffer (20 mM Tris pH 7.5, 150 mM NaCl, 1 mM EDTA) containing 1× Halt Protease and Phosphatase Inhibitor Cocktail (ThermoFisher, Waltham, MA, USA) and 10 mM NEM. Membranes were blocked in 5% milk. Antibodies were prepared in 5% BSA, as indicated in Supplemental Materials and methods. Horse Radish Peroxidase conjugated secondary antibodies (Jackson Laboratory, Bar Harbor, ME, USA) were prepared in 5% milk at a ratio of 1:10,000. Band quantification was carried out using ImageJ and normalized to β-actin [65].

### 4.7. RNA Extraction and Quantitative RT-PCR

Total RNA was harvested from BM cells using the RNeasy Kit (QIAGEN, Hilden, Germany). Following extraction, total RNA was used for cDNA synthesis, using the High Capacity RNA-to-cDNA Kit (ThermoFisher). cDNA was quantified by measuring absorbance at A280 nm, and qRT-PCR was carried out on equal concentrations of cDNA from each sample, using the iTaq Universal SYBR Green Supermix (BioRad, Hercules, CA, USA). Primers are listed in Supplemental Materials and methods.

### 4.8. TMT Labeling and Mass Spectrometry

For global proteome quantification, splenic tumor cells were isolated as described above from two to three mice per genotype. Samples were prepared and TMT-labeled per manufacturer’s protocol (ThermoFisher TMT10plex Mass Tag Labeling Kits). Following TMT labeling, acetonitrile was removed by speedvac, and samples were resuspended in 0.1% trifluoroacetic acid. Sample cleanup with Pierce^TM^ C18 tips was performed per manufacturer’s protocol (ThermoFisher). Sample concentrations were re-quantified using the Pierce^TM^ Quantitative Colorimetric Peptide Assay kit and combined in equal concentrations (ThermoFisher). Following combination, samples were dried by speedvac and fractionated by ThermoFisher high pH reverse phase fractionation kit, following manufacturer’s protocol for TMT. Resulting fractions were dried in a speedvac and resuspended in 0.1% Formic Acid for MS analysis (see Supplemental Materials and methods). Data are available via ProteomeXchange, with identifier PXD014387 [66].

### 4.9. Proteasome Activity Assay

Tumor cells from isolated spleen tissue (2 × 10^6^) were lysed in 0.5% NP-40 in PBS for 10 min on ice, with vortexing. An amount in the range 5–10 µg protein lysates was cultured and proteasome activity measured using the 20S Proteasome Activity Assay kit (APT280, Millipore, Billerica, MA, USA), per manufacturer’s protocol.

### 4.10. MTT Assay

A total of 2 × 10^5^ cells were plated into each well of a 96-well plate and cultured for 24 h in 100 µL tumor growth medium (StemSpan SFEM (Stem Cell Technologies), 5% Penn/Strep, 5% Lipid mixture, 5% Glutamate, 20 ng/mL SCF, 10 ng/mL IL-6, 10 ng/mL IL-3), containing DMSO or varying concentrations of bortezomib. Following culture, CellTiter 96 AQ_ueous_ One Solution Cell Proliferation Assay (MTS) (Promega, Madison, WI, USA) was performed per manufacturer’s protocol, using a 4 h incubation period.

### 4.11. Statistical Analysis

All experiments were performed in triplicate unless noted, and statistical analyses were performed using unpaired two-tailed Student’s *t*-test, assuming experimental samples of equal variance. For survival curve analyses, the *p*-value was calculated using a Log-rank (Mantel-cox) test. * *p*-value < 0.05, ** *p*-value < 0.01, *** *p*-value < 0.001, **** *p*-value < 0.0001.

## 5. Conclusions

AML patients frequently have reduced *FBXO9* expression that correlates with poor survival at early time-points following initial diagnosis. Analyses using a newly developed conditional *Fbxo9* KO mouse model show that loss of this gene in the hematopoietic system has very little effect on normal hematopoiesis. However, when expression is reduced in combination with expression of the AML-initiating fusion protein *Cbfb^+/56M^*, its loss leads to a decreased time of survival and an increased expansion of tumor cells in a secondary transplant. Quantitative proteomic analysis reveals that loss of *Fbxo9* corresponds with increased proteasome component expression. Tumors with reduced *Fbxo9* had increased proteasome activity and increased sensitivity to proteasome inhibition by bortezomib. Taken together, the data presented herein indicate that *FBXO9* expression could be used as a marker to identify patients with an AML, that will respond better to proteasome inhibition therapy, should front-line treatments fail to eliminate the disease.

## Supplementary Materials

The following are available online at https://www.mdpi.com/2072-6694/11/11/1717/s1, Figure S1: Pediatric patients with low *FBXO9* expression tend to have a shorter time of survival, Figure S2: Deletion of *Fbxo9* exon 4 results in a frame shift and premature stop, Figure S3: Loss of *Fbxo9* does not affect hematopoietic cell populations, Figure S4: Mice with inv(16) AML and *Fbxo9* cKO have a more aggressive and homogenous tumor, Figure S5: Mice with transplanted inv(16) tumor cells show a more aggressive phenotype for cells lacking *Fbxo9* expression, Figure S6: Western blots, Figure S7: RNA expression correlation of UPS associated proteins, Table S1: MS Supplemental Table_AML.

## References

[1] Ferrara F., Schiffer C.A. (2013). Acute myeloid leukaemia in adults. Lancet.

[2] Noone A.M., Howlader N., Krapcho M., Miller D., Brest A., Yu M., Ruhl J., Tatalovich Z., Mariotto A., Lewis D.R. (2018). SEER Cancer Statistics Review, 1975–2015.

[3] Castaigne S., Pautas C., Terre C., Raffoux E., Bordessoule D., Bastie J.N., Legrand O., Thomas X., Turlure P., Reman O. (2012). Effect of gemtuzumab ozogamicin on survival of adult patients with de-novo acute myeloid leukaemia (ALFA-0701): A randomised, open-label, phase 3 study. Lancet.

[4] Lancet J.E., Rizzieri D., Schiller G.J., Stuart R.K., Kolitz J.E., Solomon S.R., Newell L.F., Erba H.P., Uy G.L., Ryan R. (2017). Overall survival (OS) with CPX-351 versus 7+3 in older adults with newly diagnosed, therapy-related acute myeloid leukemia (tAML): Subgroup analysis of a phase III study. J. Clin. Oncol..

[5] Stein E.M., DiNardo C.D., Pollyea D.A., Fathi A.T., Roboz G.J., Altman J.K., Stone R.M., DeAngelo D.J., Levine R.L., Flinn I.W. (2017). Enasidenib in mutant IDH2 relapsed or refractory acute myeloid leukemia. Blood.

[6] Stone R.M., Mandrekar S.J., Sanford B.L., Laumann K., Geyer S., Bloomfield C.D., Thiede C., Prior T.W., Dohner K., Marcucci G. (2017). Midostaurin plus Chemotherapy for Acute Myeloid Leukemia with a FLT3 Mutation. N. Engl. J. Med..

[7] Sadowski M., Suryadinata R., Tan A.R., Roesley S.N., Sarcevic B. (2012). Protein monoubiquitination and polyubiquitination generate structural diversity to control distinct biological processes. IUBMB Life.

[8] Pham L.V., Tamayo A.T., Yoshimura L.C., Lo P., Ford R.J. (2003). Inhibition of constitutive NF-kappa B activation in mantle cell lymphoma B cells leads to induction of cell cycle arrest and apoptosis. J. Immunol..

[9] Singhal S., Mehta J., Desikan R., Ayers D., Roberson P., Eddlemon P., Munshi N., Anaissie E., Wilson C., Dhodapkar M. (1999). Antitumor activity of thalidomide in refractory multiple myeloma. N. Engl. J. Med..

[10] Metzger M.B., Hristova V.A., Weissman A.M. (2012). HECT and RING finger families of E3 ubiquitin ligases at a glance. J. Cell Sci..

[11] Kipreos E.T., Pagano M. (2000). The F-box protein family. Genome Biol..

[12] Cenciarelli C., Chiaur D.S., Guardavaccaro D., Parks W., Vidal M., Pagano M. (1999). Identification of a family of human F-box proteins. Curr. Biol..

[13] Winston J.T., Koepp D.M., Zhu C., Elledge S.J., Harper J.W. (1999). A family of mammalian F-box proteins. Curr. Biol..

[14] Moran-Crusio K., Reavie L.B., Aifantis I. (2012). Regulation of hematopoietic stem cell fate by the ubiquitin proteasome system. Trends Immunol..

[15] Strikoudis A., Guillamot M., Aifantis I. (2014). Regulation of stem cell function by protein ubiquitylation. EMBO Rep..

[16] Wang Z., Liu P., Inuzuka H., Wei W. (2014). Roles of F-box proteins in cancer. Nat. Rev. Cancer.

[17] Thompson B.J., Buonamici S., Sulis M.L., Palomero T., Vilimas T., Basso G., Ferrando A., Aifantis I. (2007). The SCFFBW7 ubiquitin ligase complex as a tumor suppressor in T cell leukemia. J. Exp. Med..

[18] Thompson B.J., Jankovic V., Gao J., Buonamici S., Vest A., Lee J.M., Zavadil J., Nimer S.D., Aifantis I. (2008). Control of hematopoietic stem cell quiescence by the E3 ubiquitin ligase Fbw7. J. Exp. Med..

[19] Reavie L., Buckley S.M., Loizou E., Takeishi S., Aranda-Orgilles B., Ndiaye-Lobry D., Abdel-Wahab O., Ibrahim S., Nakayama K.I., Aifantis I. (2013). Regulation of c-Myc ubiquitination controls chronic myelogenous leukemia initiation and progression. Cancer Cell.

[20] Vaites L.P., Lee E.K., Lian Z., Barbash O., Roy D., Wasik M., Klein-Szanto A.J., Rustgi A.K., Diehl J.A. (2011). The Fbx4 tumor suppressor regulates cyclin D1 accumulation and prevents neoplastic transformation. Mol. Cell. Biol..

[21] Chen B.B., Glasser J.R., Coon T.A., Zou C., Miller H.L., Fenton M., McDyer J.F., Boyiadzis M., Mallampalli R.K. (2012). F-box protein FBXL2 targets cyclin D2 for ubiquitination and degradation to inhibit leukemic cell proliferation. Blood.

[22] Ueda T., Nagamachi A., Takubo K., Yamasaki N., Matsui H., Kanai A., Nakata Y., Ikeda K., Konuma T., Oda H. (2015). Fbxl10 overexpression in murine hematopoietic stem cells induces leukemia involving metabolic activation and upregulation of Nsg2. Blood.

[23] Wang L., Feng W., Yang X., Yang F., Wang R., Ren Q., Zhu X., Zheng G. (2018). Fbxw11 promotes the proliferation of lymphocytic leukemia cells through the concomitant activation of NF-kappaB and beta-catenin/TCF signaling pathways. Cell Death Dis..

[24] Chen J.Y., Wang M.C., Hung W.C. (2011). Bcr-Abl-induced tyrosine phosphorylation of Emi1 to stabilize Skp2 protein via inhibition of ubiquitination in chronic myeloid leukemia cells. J. Cell. Physiol..

[25] Fernandez-Saiz V., Targosz B.S., Lemeer S., Eichner R., Langer C., Bullinger L., Reiter C., Slotta-Huspenina J., Schroeder S., Knorn A.M. (2013). SCFFbxo9 and CK2 direct the cellular response to growth factor withdrawal via Tel2/Tti1 degradation and promote survival in multiple myeloma. Nat. Cell Biol..

[26] Haferlach T., Kohlmann A., Wieczorek L., Basso G., Kronnie G.T., Bene M.C., De Vos J., Hernandez J.M., Hofmann W.K., Mills K.I. (2010). Clinical utility of microarray-based gene expression profiling in the diagnosis and subclassification of leukemia: Report from the International Microarray Innovations in Leukemia Study Group. J. Clin. Oncol..

[27] Farrar J.E., Schuback H.L., Ries R.E., Wai D., Hampton O.A., Trevino L.R., Alonzo T.A., Guidry Auvil J.M., Davidsen T.M., Gesuwan P. (2016). Genomic Profiling of Pediatric Acute Myeloid Leukemia Reveals a Changing Mutational Landscape from Disease Diagnosis to Relapse. Cancer Res..

[28] Ley T.J., Miller C., Ding L., Raphael B.J., Mungall A.J., Robertson A., Hoadley K., Triche T.J., Laird P.W., Cancer Genome Atlas Research Network (2013). Genomic and epigenomic landscapes of adult de novo acute myeloid leukemia. N. Engl. J. Med..

[29] Quadros R.M., Miura H., Harms D.W., Akatsuka H., Sato T., Aida T., Redder R., Richardson G.P., Inagaki Y., Sakai D. (2017). Easi-CRISPR: A robust method for one-step generation of mice carrying conditional and insertion alleles using long ssDNA donors and CRISPR ribonucleoproteins. Genome Biol..

[30] Kuhn R., Schwenk F., Aguet M., Rajewsky K. (1995). Inducible gene targeting in mice. Science.

[31] Liu P., Tarle S.A., Hajra A., Claxton D.F., Marlton P., Freedman M., Siciliano M.J., Collins F.S. (1993). Fusion between transcription factor CBF beta/PEBP2 beta and a myosin heavy chain in acute myeloid leukemia. Science.

[32] Kuo Y.H., Landrette S.F., Heilman S.A., Perrat P.N., Garrett L., Liu P.P., Le Beau M.M., Kogan S.C., Castilla L.H. (2006). Cbf beta-SMMHC induces distinct abnormal myeloid progenitors able to develop acute myeloid leukemia. Cancer Cell.

[33] Hyde R.K., Kamikubo Y., Anderson S., Kirby M., Alemu L., Zhao L., Liu P.P. (2010). Cbfb/Runx1 repression-independent blockage of differentiation and accumulation of Csf2rb-expressing cells by Cbfb-MYH11. Blood.

[34] Boulay P.L., Schlienger S., Lewis-Saravalli S., Vitale N., Ferbeyre G., Claing A. (2011). ARF1 controls proliferation of breast cancer cells by regulating the retinoblastoma protein. Oncogene.

[35] de Groot M., Iyer A., Zurolo E., Anink J., Heimans J.J., Boison D., Reijneveld J.C., Aronica E. (2012). Overexpression of ADK in human astrocytic tumors and peritumoral tissue is related to tumor-associated epilepsy. Epilepsia.

[36] Cui X., Liu Y., Wan C., Lu C., Cai J., He S., Ni T., Zhu J., Wei L., Zhang Y. (2014). Decreased expression of SERPINB1 correlates with tumor invasion and poor prognosis in hepatocellular carcinoma. J. Mol. Histol..

[37] Huang S., Chi Y., Qin Y., Wang Z., Xiu B., Su Y., Guo R., Guo L., Sun H., Zeng C. (2018). CAPG enhances breast cancer metastasis by competing with PRMT5 to modulate STC-1 transcription. Theranostics.

[38] Lai C.Y., Liu H., Tin K.X., Huang Y., Yeh K.H., Peng H.W., Chen H.D., He J.Y., Chiang Y.J., Liu C.S. (2019). Identification of UAP1L1 as a critical factor for protein O-GlcNAcylation and cell proliferation in human hepatoma cells. Oncogene.

[39] Ma X., Qi W., Pan H., Yang F., Deng J. (2018). Overexpression of USP5 contributes to tumorigenesis in non-small cell lung cancer via the stabilization of beta-catenin protein. Am. J. Cancer Res..

[40] Wang J.H., Yuan L.J., Liang R.X., Liu Z.G., Li B.H., Wen Z.S., Huang S.T., Zheng M. (2017). GOLPH3 promotes cell proliferation and tumorigenicity in esophageal squamous cell carcinoma via mTOR and Wnt/betacatenin signal activation. Mol. Med. Rep..

[41] Yang X.Y., Liao J.J., Xue W.R. (2019). FMNL1 down-regulation suppresses bone metastasis through reducing TGF-beta1 expression in non-small cell lung cancer (NSCLC). Biomed. Pharmacother..

[42] Zhang Y., Zou X., Qian W., Weng X., Zhang L., Zhang L., Wang S., Cao X., Ma L., Wei G. (2019). Enhanced PAPSS2/VCAN sulfation axis is essential for Snail-mediated breast cancer cell migration and metastasis. Cell Death Differ..

[43] Chen D., Dou Q.P. (2010). The ubiquitin-proteasome system as a prospective molecular target for cancer treatment and prevention. Curr. Protein Peptied Sci..

[44] Betz B.L., Hess J.L. (2010). Acute myeloid leukemia diagnosis in the 21st century. Arch. Pathol. Lab. Med..

[45] George J., Uyar A., Young K., Kuffler L., Waldron-Francis K., Marquez E., Ucar D., Trowbridge J.J. (2016). Leukaemia cell of origin identified by chromatin landscape of bulk tumour cells. Nat. Commun..

[46] Arlt A., Bauer I., Schafmayer C., Tepel J., Muerkoster S.S., Brosch M., Roder C., Kalthoff H., Hampe J., Moyer M.P. (2009). Increased proteasome subunit protein expression and proteasome activity in colon cancer relate to an enhanced activation of nuclear factor E2-related factor 2 (Nrf2). Oncogene.

[47] Chen L., Madura K. (2005). Increased proteasome activity, ubiquitin-conjugating enzymes, and eEF1A translation factor detected in breast cancer tissue. Cancer Res..

[48] Stoebner P.E., Lavabre-Bertrand T., Henry L., Guiraud I., Carillo S., Dandurand M., Joujoux J.M., Bureau J.P., Meunier L. (2005). High plasma proteasome levels are detected in patients with metastatic malignant melanoma. Br. J. Dermatol..

[49] Tsvetkov P., Adler J., Myers N., Biran A., Reuven N., Shaul Y. (2018). Oncogenic addiction to high 26S proteasome level. Cell Death Dis..

[50] Ma W., Kantarjian H., Zhang X., Wang X., Estrov Z., O’Brien S., Albitar M. (2011). Ubiquitin-proteasome system profiling in acute leukemias and its clinical relevance. Leuk. Res..

[51] Fowler N., Kahl B.S., Lee P., Matous J.V., Cashen A.F., Jacobs S.A., Letzer J., Amin B., Williams M.E., Smith S. (2011). Bortezomib, bendamustine, and rituximab in patients with relapsed or refractory follicular lymphoma: The phase II VERTICAL study. J. Clin. Oncol..

[52] Moreau P., Richardson P.G., Cavo M., Orlowski R.Z., San Miguel J.F., Palumbo A., Harousseau J.L. (2012). Proteasome inhibitors in multiple myeloma: 10 years later. Blood.

[53] Robak T., Huang H., Jin J., Zhu J., Liu T., Samoilova O., Pylypenko H., Verhoef G., Siritanaratkul N., Osmanov E. (2015). Bortezomib-based therapy for newly diagnosed mantle-cell lymphoma. N. Engl. J. Med..

[54] Sarlo C., Buccisano F., Maurillo L., Cefalo M., Di Caprio L., Cicconi L., Ditto C., Ottaviani L., Di Veroli A., Del Principe M.I. (2013). Phase II Study of Bortezomib as a Single Agent in Patients with Previously Untreated or Relapsed/Refractory Acute Myeloid Leukemia Ineligible for Intensive Therapy. Leuk. Res. Treat..

[55] Attar E.C., Johnson J.L., Amrein P.C., Lozanski G., Wadleigh M., DeAngelo D.J., Kolitz J.E., Powell B.L., Voorhees P., Wang E.S. (2013). Bortezomib added to daunorubicin and cytarabine during induction therapy and to intermediate-dose cytarabine for consolidation in patients with previously untreated acute myeloid leukemia age 60 to 75 years: CALGB (Alliance) study 10502. J. Clin. Oncol..

[56] Blum W., Schwind S., Tarighat S.S., Geyer S., Eisfeld A.K., Whitman S., Walker A., Klisovic R., Byrd J.C., Santhanam R. (2012). Clinical and pharmacodynamic activity of bortezomib and decitabine in acute myeloid leukemia. Blood.

[57] Horton T.M., Perentesis J.P., Gamis A.S., Alonzo T.A., Gerbing R.B., Ballard J., Adlard K., Howard D.S., Smith F.O., Jenkins G. (2014). A Phase 2 study of bortezomib combined with either idarubicin/cytarabine or cytarabine/etoposide in children with relapsed, refractory or secondary acute myeloid leukemia: A report from the Children’s Oncology Group. Pediatr. Blood Cancer.

[58] Walker A.R., Wang H., Walsh K., Bhatnagar B., Vasu S., Garzon R., Canning R., Geyer S., Wu Y.Z., Devine S.M. (2016). Midostaurin, bortezomib and MEC in relapsed/refractory acute myeloid leukemia. Leuk. Lymphoma.

[59] Warlick E.D., Cao Q., Miller J. (2013). Bortezomib and vorinostat in refractory acute myelogenous leukemia and high-risk myelodysplastic syndromes: Produces stable disease but at the cost of high toxicity. Leukemia.

[60] Orlowski R.Z., Voorhees P.M., Garcia R.A., Hall M.D., Kudrik F.J., Allred T., Johri A.R., Jones P.E., Ivanova A., Van Deventer H.W. (2005). Phase 1 trial of the proteasome inhibitor bortezomib and pegylated liposomal doxorubicin in patients with advanced hematologic malignancies. Blood.

[61] Guzman M.L., Swiderski C.F., Howard D.S., Grimes B.A., Rossi R.M., Szilvassy S.J., Jordan C.T. (2002). Preferential induction of apoptosis for primary human leukemic stem cells. Proc. Natl. Acad. Sci. USA.

[62] Kagoya Y., Yoshimi A., Kataoka K., Nakagawa M., Kumano K., Arai S., Kobayashi H., Saito T., Iwakura Y., Kurokawa M. (2014). Positive feedback between NF-kappaB and TNF-alpha promotes leukemia-initiating cell capacity. J. Clin. Investig..

[63] San Miguel J., Blade J., Boccadoro M., Cavenagh J., Glasmacher A., Jagannath S., Lonial S., Orlowski R.Z., Sonneveld P., Ludwig H. (2006). A practical update on the use of bortezomib in the management of multiple myeloma. Oncologist.

[64] Aplenc R., Meshinchi S., Sung L., Alonzo T.A., Pollard J., Gerbing R.B., Raimondi S.C., Hirsch B.A., Loken M.R., Winter L. (2016). The Addition of Bortezomib to Standard Chemotherapy for Pediatric Acute Myeloid Leukemia Has Increased Toxicity without Therapeutic Benefit: A Report from the Children’s Oncology Group.

[65] Schneider C.A., Rasband W.S., Eliceiri K.W. (2012). NIH Image to ImageJ: 25 years of image analysis. Nat. Methods.

[66] Deutsch E.W., Csordas A., Sun Z., Jarnuczak A., Perez-Riverol Y., Ternent T., Campbell D.S., Bernal-Llinares M., Okuda S., Kawano S. (2017). The ProteomeXchange consortium in 2017: Supporting the cultural change in proteomics public data deposition. Nucleic Acids Res..

