# Supplementary material for: Loss of FBXO9 Enhances Proteasome Activity and Promotes Aggressiveness in Acute Myeloid Leukemia
Source: Cancers (Basel). 2019 Nov 3;11(11):1717. doi: 10.3390/cancers11111717 (PMC6895989; doi:10.3390/cancers11111717)
Supplement: Supplementary file 1 [file cancers-11-01717-s001.pdf]

Supplemental Materials and Methods

Table S1. MS Supplemental Table_AML.

Genotyping Primer	Sequence
Fbxo9 5′ F	5′ – GTC TCT TCG AGG CAG AAC GTA TC – 3′
Fbxo9 5′ R	5′ – GCA ACG GGA TCA GAT GTC CAA TG – 3′
Fbxo9 3′ F	5′ – GCC TGA GAC TGA AAG ATC CTT GC – 3′
Fbxo9 3′ R	5′ – CAG GCT TAC AAA GAG GGC CTA CG – 3′
Hygro F	5′ – CCA TCG TCG AGA TCC AGA CAT – 3′
Hygro R	5′ – GTA TAT GCT CCG CAT TGG TCT TG – 3′
Generic Cre F	5′ – GCG GTC TGG CAG TAA AAA CTA TC – 3′
Generic Cre R	5′ – GTG AAA CAG CAT TGC TGT CAC TT – 3′
Inv Post F	5′ – CCC CCG TAC TGT GTG TGT CT – 3′
Inv Post R	5′ – GCC AGA CGG GTC AAC AAT AC – 3′

qRT-PCR Primer	Sequence
Gapdh F	5′ – CAT GGC CTT CCG TGT TCC TA – 3′
Gapdh R	5′ – CTG GTC CTC AGT GTA GCC CAA – 3′
Fbxo9 Exon 4 F	5′ – AGA AGC TCT ATG CTG AAA GCA G – 3′
Fbxo9 Exon 4 R	5′ – CAT CGC CCT ACG GTA GAA CT – 3′
Fbxo9 Exon 2-3 F	5′ – ATG AGA GTC CGG CTG AGA GA – 3′
Fbxo9 Exon 2-3 R	5′ – AGA GCT TCT TCC TGC TCT GC – 3′

Western Blot Antibodies:

Antibody	Company	Ref#
ARF-1	Proteintech	10790-1-AP
B-actin-HRP	Santa Cruz	sc-47778
FLAG-HRP	Proteintech	12926-1-AP
PSMA2	Bethyl	A303-816A
PSMB7	Bethyl	A303-848A
PSMD11	Cell Signaling	14303S

Flow Cytometry Antibodies

Antibody	Fluorochrome	Clone	Company
7-AAD	n/a	n/a	eBioscience
Annexin V	PE	n/a	BioLegend
B220	PacBlue, BV510, APCcy7, APC, Biotin, PE	RA3-6B2	BioLegend
CD4	APCcy7, APC, Biotin, PE	CK1.5	BioLegend
CD8	APCcy7, APC, Biotin, PE	53-6.7	BioLegend, eBioscience
CD11b	FITC, APCcy7, APC, Biotin, PE	M1/70	BioLegend
CD16/32	FITC, APC	93	BioLegend
CD34	APC, PE	HM34	BioLegend
CD48	FITC	HM48-1	BioLegend
CD131	PE	n/a	BD Pharmingen
CD150	BV510, BV785	TC15-12F12.2	BioLegend
cKit	APC, BV421, PacBlue	2B8	BioLegend
Gr-1	APCcy7, APC, Biotin, PE, BV421	RB6-8C5	BioLegend
Ki67	FITC	16A8	BioLegend
Sca-1	PE, PEcy7	D7	BioLegend, eBioscience
Strep	FITC	n/a	BioLegend
Ter-119	APCcy7, PacBlue, APC, Biotin, PE	TER-119	BioLegend

Class	MILE Study Diagnosis	No of Samples
HBM	Non-leukemia and healthy bone marrow	74
MDS	Myelodisplastic syndrome	206
Normal	AML with normal karyotype	351
Complex	AML with complex karyotype	48
inv(16)	AML with inv(16)/t(16;16)	28
t(15;17)	AML with t(15;17)	37
t(8;21)	AML with t(8;21)	40
MLL	AML with t(11q23)/MLL	38
CML	Chronic myeloid leukemia	76
Total		898

Class	TARGET Study Diagnosis	No of Samples
HBM	Non-leukemia and healthy bone marrow	5
Unknown	AML with unknown diagnosis	10
Normal	AML with normal karyotype	53
inv(16)	AML with inv(16)/t(16;16)	36
t(8;21)	AML with t(8;21)	45
MLL	AML with t(11q23)/MLL	41
Other	AML with other diagnoses	47
Total		237

FAB Subtype	TCGA Study Diagnosis	No of Samples
AML M0	AML with minimal maturation	15
AML M1	AML without maturation	41
AML M2	AML with maturation	42
AML M3	Acute promyelocytic leukemia	15
AML M4	Acute myelomonocytic leukemia	37
AML M5	Acute monoblastic or monocytic leukemia	22
AML M6	Acute erythroid leukemia	2
AML M7	Acute megakaryoblastic leukemia	3
Other	Other subtype	2
Total		179

Mass Spectrometry Method:

Samples were loaded onto trap column Acclaim PepMap 100 75 μm x 2 cm C18 LC Columns (Thermo Scientific™) at flow rate of 5 μl/min then separated with a Thermo RSLC Ultimate 3000 (Thermo Scientific™) from 5-20% solvent B (0.1% FA in 80% ACN) from 10-98 minutes at 300 nL/min and 50 ºC with a 120 minutes total run time for fractions one and two. For fractions three to six, solvent B was used at 5-45% for the same duration. Eluted peptides were analyzed by a Thermo Orbitrap Fusion Lumos Tribrid (Thermo Scientific™) mass spectrometer in a data dependent acquisition mode using synchronous precursor selection method. A survey full scan MS (from m/z 375-1500) was acquired in the Orbitrap with a resolution of 120000. The AGC target for MS2 in iontrap was set as 1×10^4 and ion filling time set as 150ms and fragmented using CID fragmentation with 35% normalized collision energy. The AGC target for MS3 in orbitrap was set as 1×10^5 and ion filling time set as 200 ms with a scan range of 100-500 and fragmented using HCD with 65% normalized collision energy. Protein identification was performed using proteome discoverer software version 2.2 (Thermo Fisher Scientific) by searching MS/MS data against the UniProt mouse protein database. The search was set up for full tryptic peptides with a maximum of 2 missed cleavage sites. Oxidation, TMT6plex of the amino terminus, GG and GGQ ubiquitination, phosphorylation, and acetylation were included as variable modifications and carbamidomethylation and TMT6plex of the amino terminus were set as fixed modifications. The precursor mass tolerance threshold was set at 10 ppm for a maximum fragment mass error of 0.6 Da with a minimum peptide length of 6 and a maximum peptide length of 144. The significance threshold of the ion score was calculated based on a false

discovery rate calculated using the percolator node. Protein accessions were put into Ingenuity Pathway Analysis (QIAGEN Inc.) to identify gene symbols and localizations. Gene ontology pathway analysis was performed using DAVID Bioinformatics Database 6.8 using the functional annotation tool.

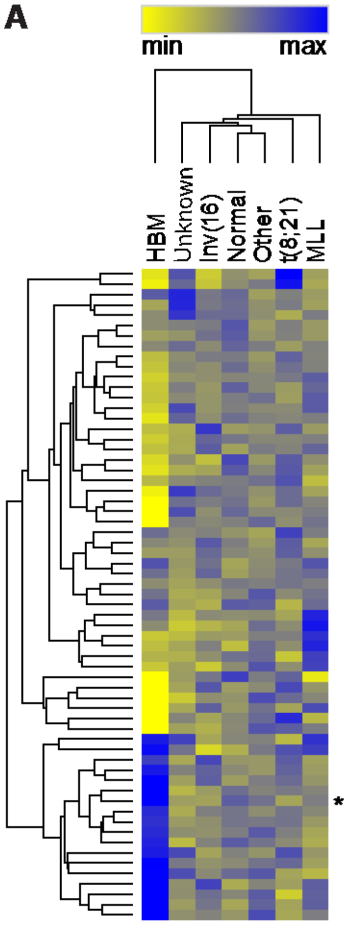

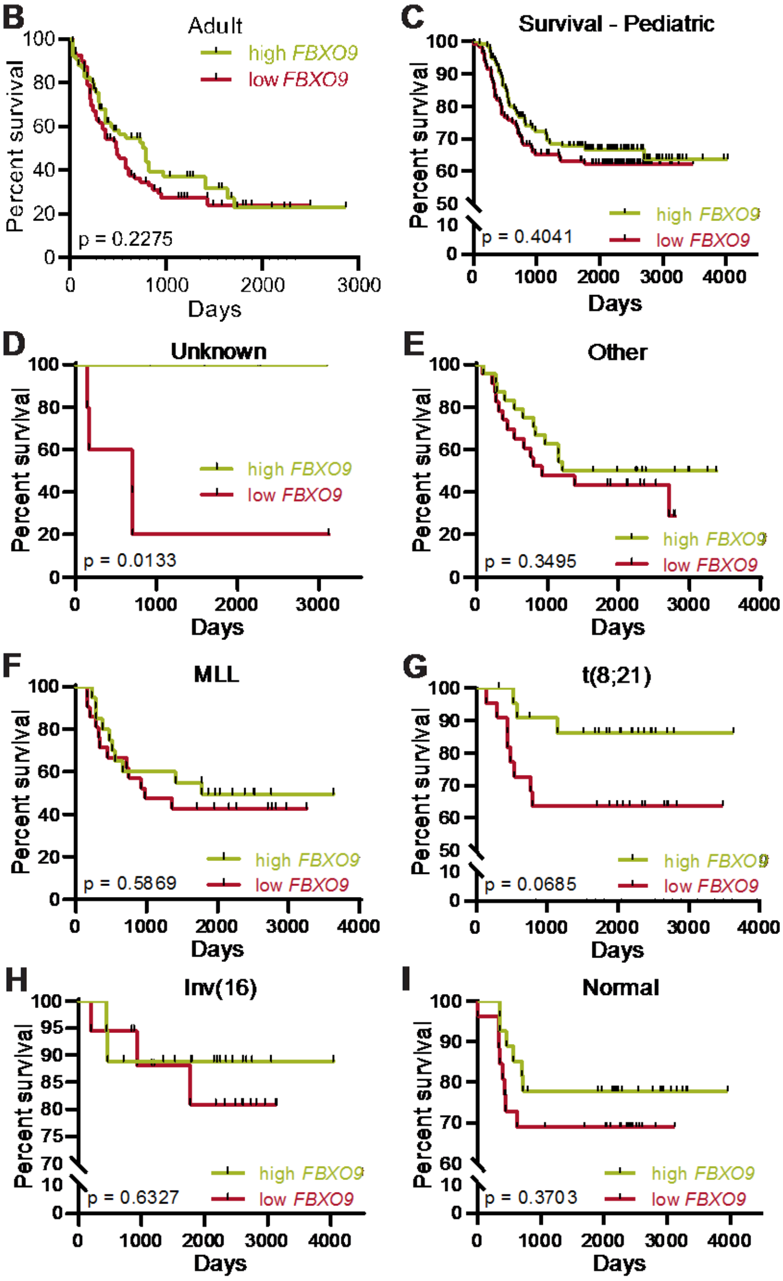

Supplementary Figure S1 Pediatric patients with low *FBXO9* expression tend to have a shorter time of survival. (A) Relative expression of F-box proteins in pediatric AML patients compared to healthy bone marrow (HBM), * denotes *FBXO9*. (B) Survival of adult patients up to 3000 days post-diagnosis. (C) Survival of pediatric patients up to 4000 days post-diagnosis. (D-H) Survival of pediatric patients of various subtypes grouped by *FBXO9* expression above (high) or below (low) the median.

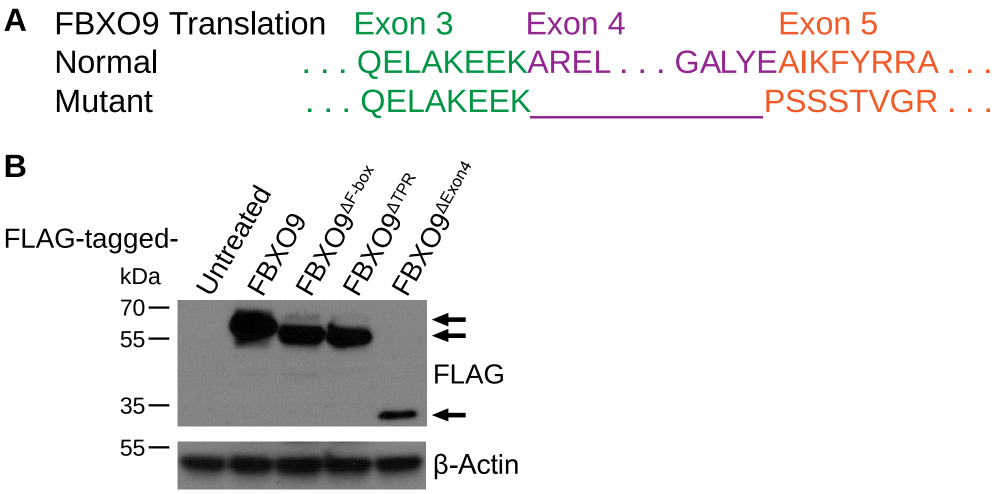

Supplementary Figure S2 Deletion of *Fbxo9* exon 4 results in a frame shift and premature stop. (A) Amino acid translation of sequenced cDNA from *Fbxo9*$^{+/+}$ and *Fbxo9*$^{-/-}$ mice. (B) Western blot expression of overexpressed flag-tagged FBXO9 with various deletion mutations.

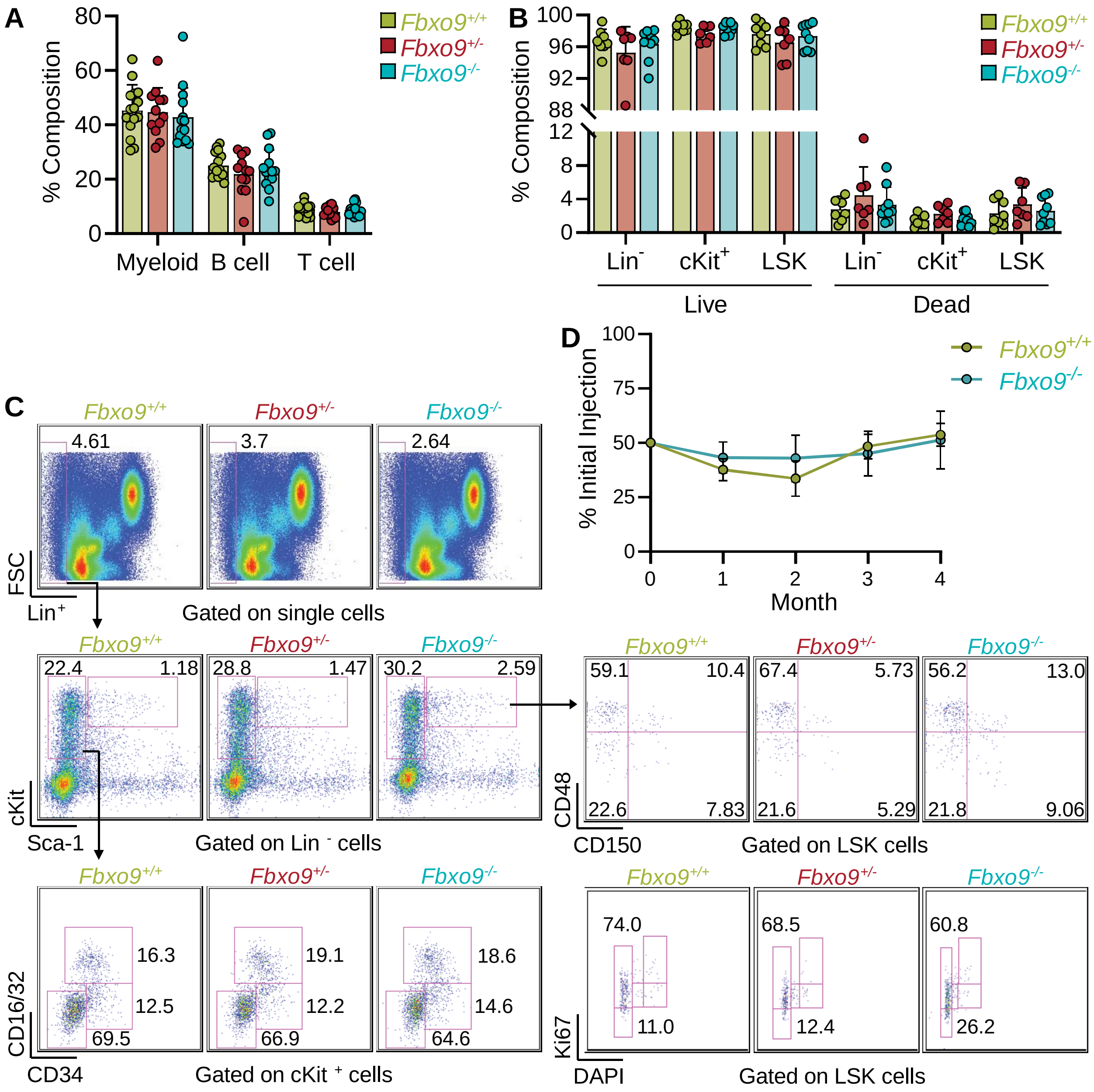

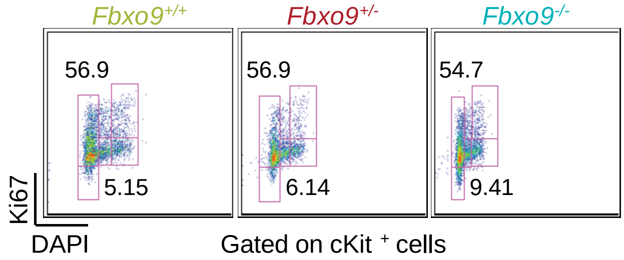

Supplementary Figure S3 Loss of *Fbxo9* does not affect hematopoietic cell populations. (A) Bar graph (mean ± standard deviation) of BM cell percentages of mature lineages following treatment with Poly(I:C). (B) Bar graph (mean ± standard deviation) of live and dead cells. (C) Flow cytometry gating strategy for isolating LSK and cKit⁺ populations from total BM and analyzing for cell cycle stage. (D) Competitive repopulation cell composition of *Fbxo9*$^{+/+}$ and *Fbxo9*$^{-/-}$ cells.

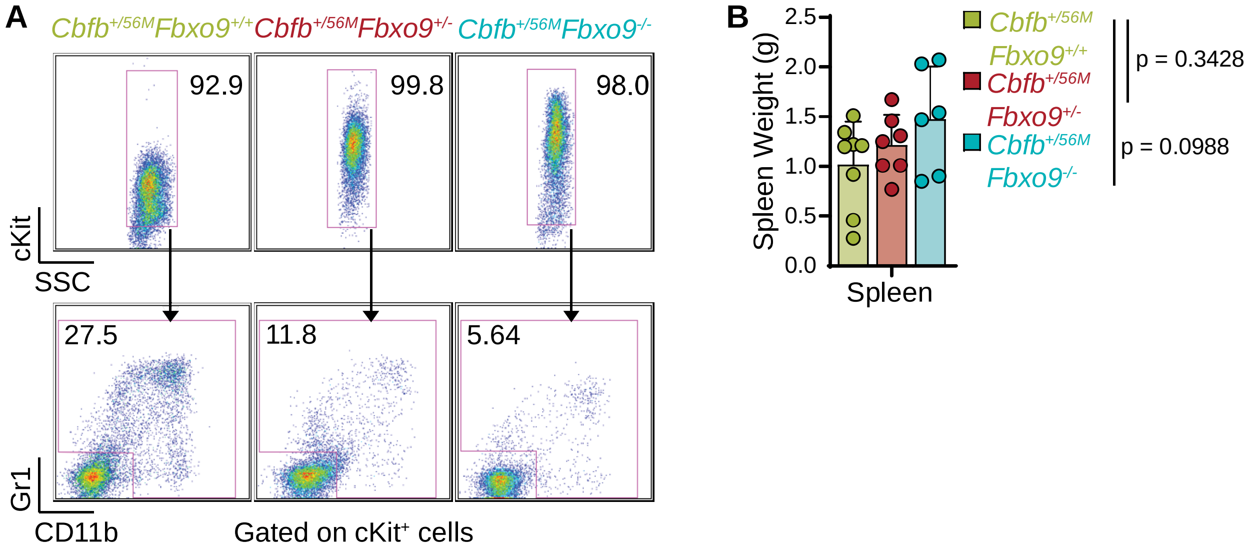

Supplementary Figure S4 Mice with inv(16) AML and *Fbxo9* cKO have a more aggressive and homogenous tumor. (A) Representative FACS plots of tumor cells isolated from the BM of mice at time of sacrifice. Cells are first gated on total live singlets and subsequently on cKit $^{+}$ cells which are further analyzed for expression of Gr1/CD11b. (B) Bar graph (mean ± standard deviation) of spleen weight of mice at time of sacrifice.

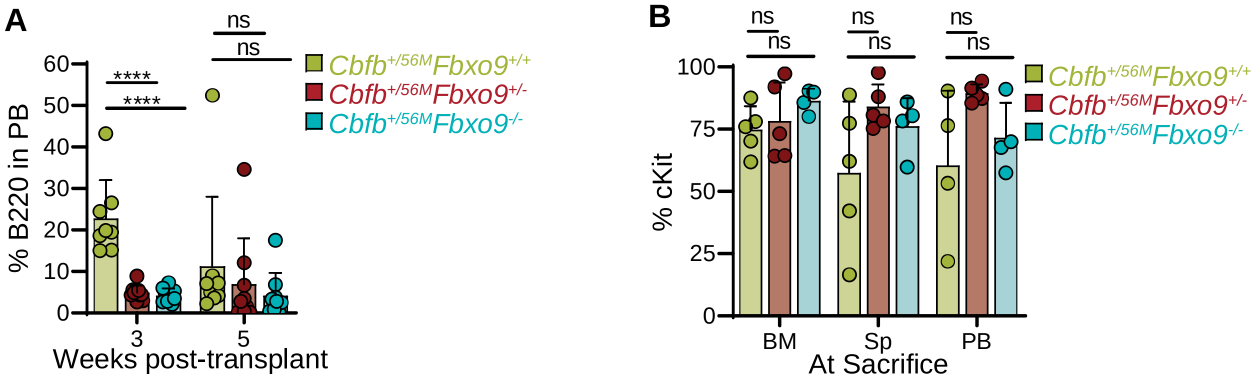

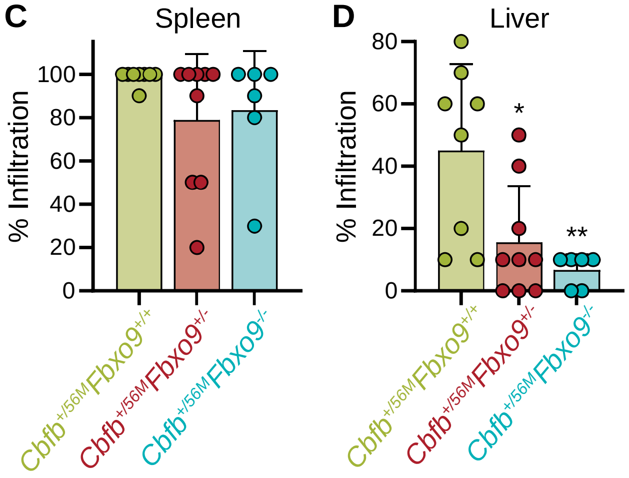

Supplementary Figure S5 Mice with transplanted inv(16) tumor cells show a more aggressive phenotype for cells lacking *Fbxo9* expression. (A) Bar graph of B cell (B220) expression in the PB following transplantation (mean ± standard deviation). (B) Bar graph of percentage of cKit^{+} tumor cells in the BM, spleen (Sp), and PB at time of sacrifice (mean ± standard deviation, **** $p < 0.0001$). (C-D) Bar graph of (C) spleen (*Cbfb*$^{+/56M}$*Fbxo9*$^{+/-}$, $p = 0.0890$ and *Cbfb*$^{+/56M}$*Fbxo9*$^{-/-}$, $p = 0.1356$) and (D) liver infiltration where each datapoint represents an individual mouse (* $p < 0.05$, ** $p < 0.01$).

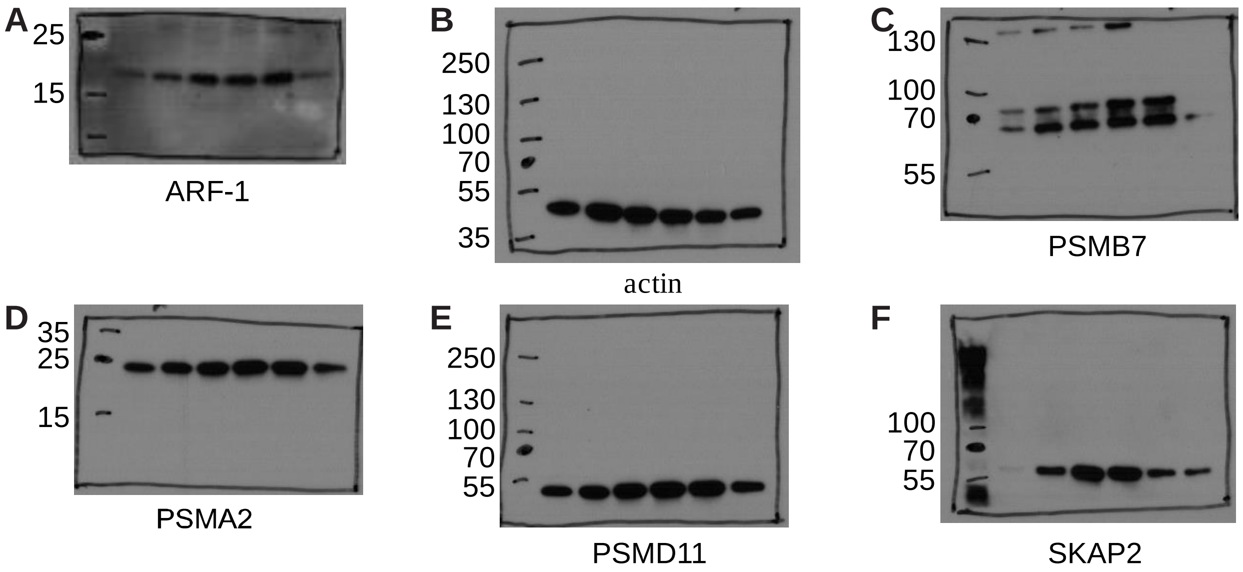

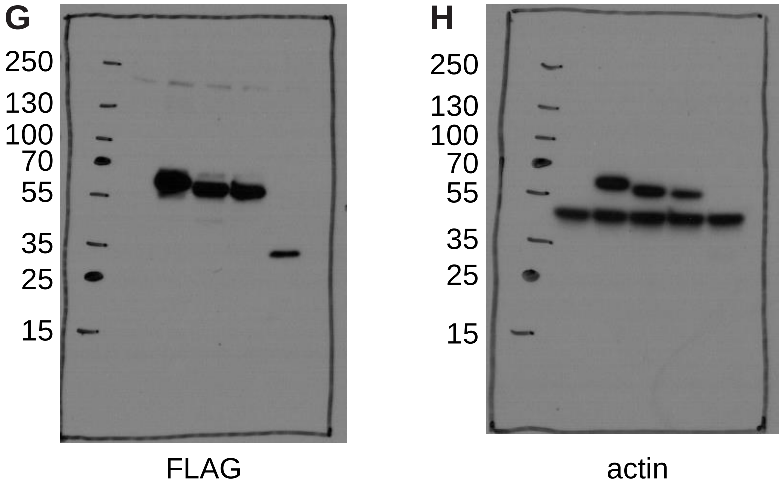

Supplementary Figure S6 Western blots. (A-F) Complete western blots corresponding to Figure 7B. (G-H) Complete western blots corresponding to Figure S2B.

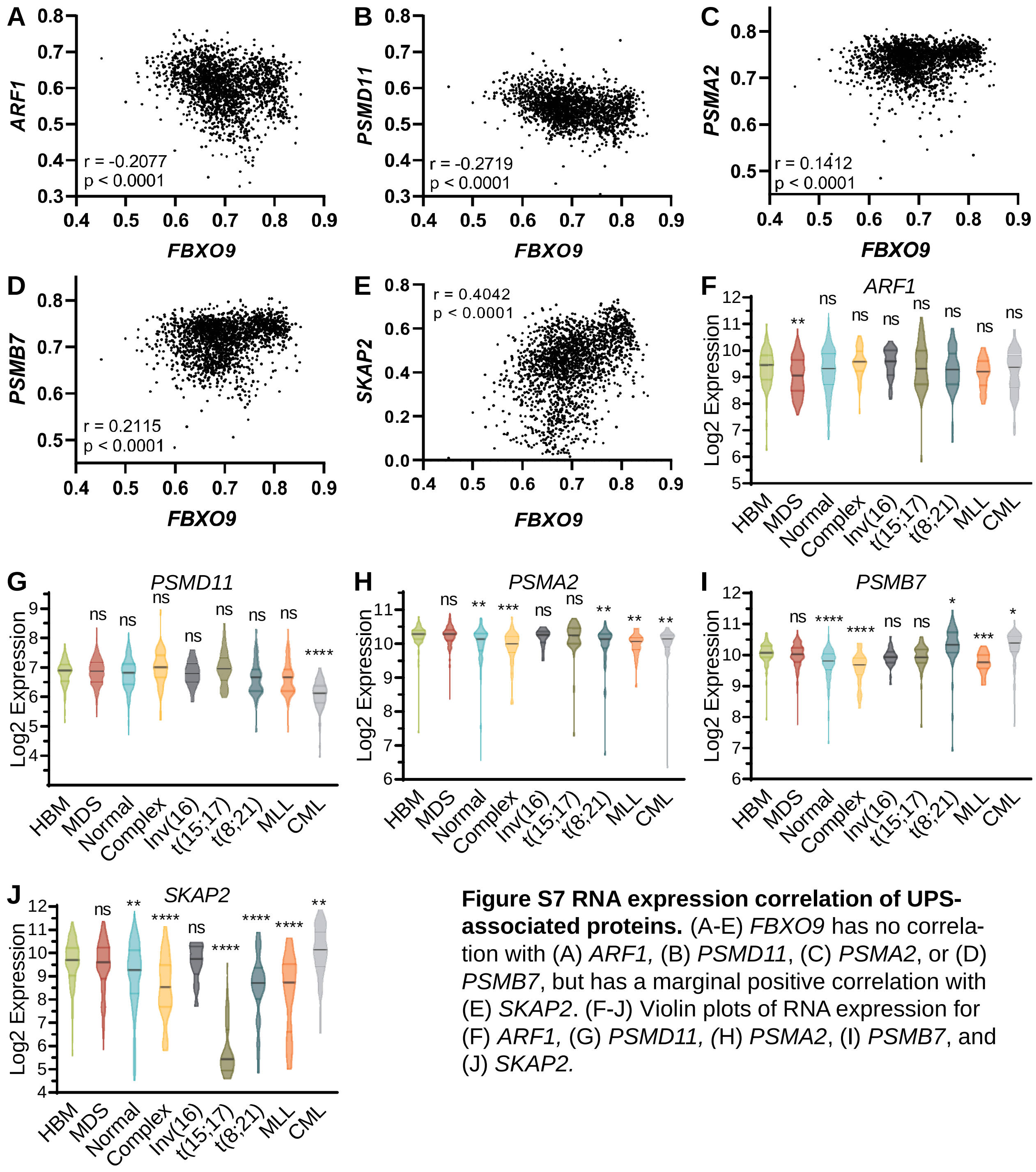

Figure S7 RNA expression correlation of UPS-associated proteins. (A-E) *FBXO9* has no correlation with (A) *ARF1,* (B) *PSMD11*, (C) *PSMA2*, or (D) *PSMB7*, but has a marginal positive correlation with (E) *SKAP2*. (F-J) Violin plots of RNA expression for (F) *ARF1,* (G) *PSMD11,* (H) *PSMA2*, (I) *PSMB7*, and (J) *SKAP2.*
